# Structure and functional analysis of a bacterial adhesin sugar-binding domain

**DOI:** 10.1371/journal.pone.0220045

**Published:** 2019-07-23

**Authors:** Tyler D. R. Vance, Shuaiqi Guo, Shayan Assaie-Ardakany, Brigid Conroy, Peter L. Davies

**Affiliations:** Department of Biomedical and Molecular Science, Queen’s University, Kingston, Ontario, Canada; University of Queensland, AUSTRALIA

## Abstract

Bacterial adhesins attach their hosts to surfaces through one or more ligand-binding domains. In RTX adhesins, which are localized to the outer membrane of many Gram-negative bacteria via the type I secretion system, we see several examples of a putative sugar-binding domain. Here we have recombinantly expressed one such ~20-kDa domain from the ~340-kDa adhesin found in *Marinobacter hydrocarbonoclasticus*, an oil-degrading bacterium. The sugar-binding domain was purified from *E*. *coli* with a yield of 100 mg/L of culture. Circular dichroism analysis showed that the protein was rich in beta-structure, was moderately heat resistant, and required Ca^2+^ for proper folding. A crystal structure was obtained in Ca^2+^ at 1.2-Å resolution, which showed the presence of three Ca^2+^ ions, two of which were needed for structural integrity and one for binding sugars. Glucose was soaked into the crystal, where it bound to the sugar’s two vicinal hydroxyl groups attached to the first and second (C1 and C2) carbons in the pyranose ring. This attraction to glucose caused the protein to bind certain polysaccharide-based column matrices and was used in a simple competitive binding assay to assess the relative affinity of sugars for the protein’s ligand-binding site. Fucose, glucose and N-acetylglucosamine bound most tightly, and N-acetylgalactosamine hardly bound at all. Isothermal titration calorimetry was used to determine specific binding affinities, which lie in the 100-μM range. Glycan arrays were tested to expand the range of ligand sugars assayed, and showed that *Mh*PA14 bound preferentially to branched polymers containing terminal sugars highlighted as strong binders in the competitive binding assay. Some of these binders have vicinal hydroxyl groups attached to the C3 and C4 carbons that are sterically equivalent to those presented by the C1 and C2 carbons of glucose.

## Introduction

Many bacterial survival strategies rely on the organisms’ ability to adhere themselves to surfaces [1–3]. In this state, they can remain in favourable, high-nutrient zones of their environment for an extended time, without substantial energy costs. Adhesion proteins (or adhesins) play a crucial part in fostering contacts between bacteria and their substrates [4,5]. Within this broad category of proteins exists a family of long, repetitive, and surface-exposed proteins called Repeats-In-Toxin adhesion proteins, or RTX adhesins [6]. While many examples of RTX adhesins exist in the NCBI database, very few of them have been functionally or structurally characterized. Those that have–including LapA and LapF from plant-associated *Pseudomonas* species [4,7–9], *Mp*IBP from the Antarctic-isolated *Marinomonas primoryensis* [10,11], and SiiE and FrhA from the pathogens *Salmonella enterica* and *Vibrio cholerae*, respectively [12–14]–facilitate interactions between the host bacterium and either an environmental surface (adhesion), or another bacterium (cohesion). In many cases, these contacts can serve as the foundation for structured, surface-associated communities–called biofilms–often composed of more than one species of microorganisms [15–17].

**Fig 1:**
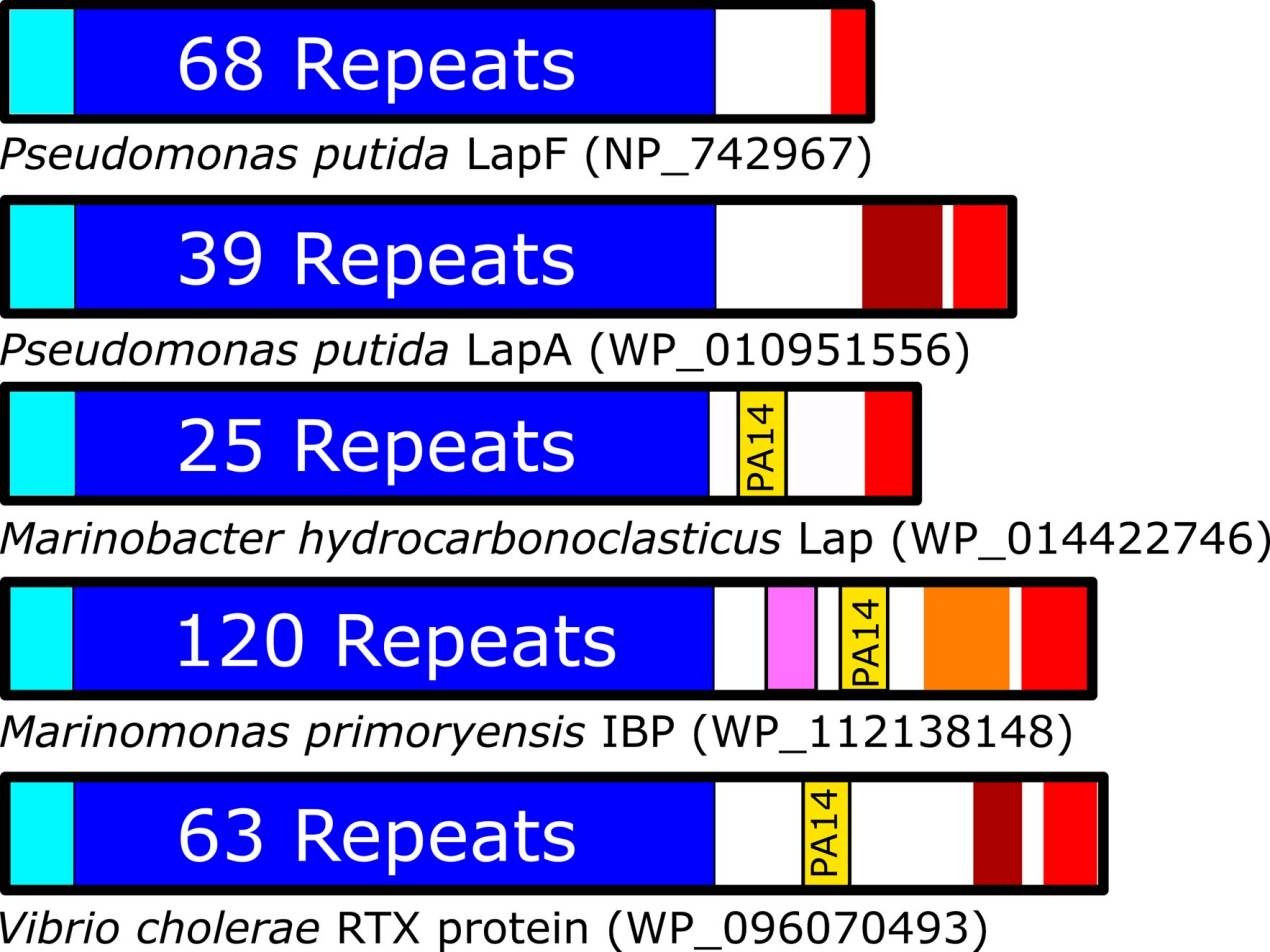
Organization of adhesins with and without PA14 domains. Domain architectures are shown for several RTX adhesins, oriented with N termini on the left. Colour scheme: cyan = bacterial membrane anchoring domains, blue = region of tandem repeat domains, yellow = PA14 domain, burgundy = von Willebrand Factor A-like domain, pink = Vibrio-like peptide-binding domain, orange = ice-binding domain, red = Type 1 Secretion Signal and RTX repeats, white = regions of unknown/unpredicted composition.

RTX adhesins are multi-domain proteins with a similar architecture (Fig 1) [6]. Their domain arrangement can be broadly split into three regions: 1) an N-terminal segment that attaches the protein to the surface of the bacterium [18,19]; 2) an extender region comprised of varying numbers of tandem repeats, which often fold as immunoglobulin-like beta sandwiches [20,21]; and 3) a C-terminal region that houses both a collection of adhesion domains that are responsible for making contact with a specific substrate, as well as the sequences required for export via the Type 1 Secretion System (T1SS) [8,22,23]. Comparing the five RTX adhesins in Fig 1, it becomes apparent that the C-terminal region has the most diversity between species, with permutations of domains capable of binding sugars, peptides, or rarer substrates like ice. Many of the C-terminal segments contain sequences of unknown structure and/or function that might include novel adhesion domains. The diversity of adhesion proteins has attracted increasing interest for further research, as examples of protein-mediated interactions between bacteria and vastly different substrates continue to emerge [11].

One adhesion domain that appears often within RTX adhesins is the PA14 domain. This domain is named after its position in the Protective Antigen (PA) from the human pathogen *Bacillus anthracis*, where it was first discovered [24]. It is now known that the PA14 domain is a component of many proteins, both prokaryotic and eukaryotic, where it appears to consistently serve a role in carbohydrate-binding [25]. One of the best-studied examples is the epithelial adhesin (EpA) family of proteins from the pathogenic yeast *Candida glabrata*. The N-terminal domain of these yeast adhesins are PA14 domains that take on a characteristic beta sandwich-like fold. Structures for the PA14 domains from EpA adhesins 1, 6, and 9 (PDB: **4ASL**, **4COY**, & **4CP0**) show a conserved calcium ion, coordinated via dual aspartate residues tandemly connected by a cis-peptide bond (D-cis-D motif) [26,27]. The calcium ion, in turn, coordinates carbohydrate hydroxyl groups, thus facilitating yeast colonization of mammalian surfaces and subsequent biofilm formation[28–30].

Given the PA14 domain’s known role in the pathogenesis of yeast, its presence in bacteria at the distal end of many RTX adhesins is of interest. At present, the only RTX adhesin PA14 domain to have been structurally and functionally characterized is that of *Mp*IBP, where it was found to foster contacts to the microalgal species *Chaetoceros neogracile* [11]. With only a single characterized example, very little can be speculated regarding the domains’ affinities for specific sugars in polysaccharides, and how these affinities could factor into the adhesin’s biological function. The following study was undertaken to characterize another RTX adhesin’s PA14 domain, namely that from the *Marinobacter hydrocarbonoclasticus* long adhesion protein (*Mh*Lap) [31,32]. *M*. *hydrocarbonoclasticus* is an oil-eating bacterium that forms biofilms at the oil-water interface to improve the bioavailability of its preferred carbon source: medium- to long-chain alkanes [33–35]. Considering the species’ potential as a bioremediation source, attaining a better understanding of the *Mh*Lap adhesin’s ability to interact with sugars–possibly for the benefit of its oleolytic biofilms–is of particular interest [36,37]. Through our study, we have elucidated the structure of recombinant *Mh*PA14 and shown it to be a calcium-dependent sugar-binding domain with affinity for dextran-based polymers. We have exploited this affinity to construct a resin-based competitive binding assay for comparing affinities to different sugars, which shows that the domain has a broad range of monosaccharide binding partners (such as fucose, glucose, N-acetylglucosamine and mannose), but is unable to bind strongly to sugars like galactose or N-acetylgalactosamine.

## Materials and methods

Proteins referenced heavily throughout the manuscript are detailed in Table 1.

**Table 1:** Accession identification for referenced proteins.

*Mh*Lap	NCBI: **WP_014422746**	Uniprot: **H8W6K8**
*Mp*IBP	NCBI: **WP_112138148**	Uniprot: **A0A2Z4PSB8**
LapA	NCBI: **WP_010951556**	Uniprot: **Q88RG2**
LapF	NCBI: **NP_742967**	Uniprot: **Q88PP2**
Vibrio RTX	NCBI: **WP_096070493**	Uniprot: N/A
EpA1	NCBI: **ALG76051**	Uniprot: **A0A0N9M4X0**

### Molecular cloning, and protein expression of *Mh*PA14

A gene encoding *Mh*PA14 was synthesized with optimal codon usage for *Escherichia coli* (GeneArt). The gene was cut with the restriction enzymes *Nde*I (5ʹ end) and *Xho*I (3ʹ end) for ligation into the pET28a expression vector, which provides an N-terminal His tag. The plasmid was then transformed into Top10 cells for plasmid amplification, and then electroporated into BL21(DE3) cells for protein expression.

*Mh*PA14 protein (sequence shown in S1 Fig) was expressed in the following manner. Single colonies were picked into 25-mL cultures of LB Broth + 0.1 mg/mL kanamycin and grown overnight at 37°C. Overnight cultures were used to inoculate 1-L cultures, which were then grown at 37°C until an OD_600_ of 0.9 was attained. IPTG was then added to a final concentration of 1 mM to induce protein expression at 23°C, and the culture was kept growing overnight.

### Purification of *Mh*PA14

*E*. *coli* cultures expressing *Mh*PA14 were spun down at 4500 x g in a JS-4.2 rotor (Beckman Coulter). The medium was discarded, and the pellet resuspended in Ni Buffer (50 mM Tris-HCl pH 9.0, 500 mM NaCl, 2 mM CaCl_2_, 5 mM imidazole) along with a protease inhibitor cocktail tablet (Roche). Cells were then lysed by sonication, and the resulting cell debris removed via centrifugation at ~30000 ×g in a JA-25.5 rotor (Beckman Coulter). The lysate supernatant was incubated with Ni-NTA Agarose Resin (Qiagen) and washed with ~ 100 mL of Ni Buffer. The bound *Mh*PA14 was then eluted with Ni Buffer supplemented with 400 mM imidazole. Eluted fractions were pooled and subjected to anion-exchange chromatography on a Q Sepharose Fast Flow column (GE Healthcare), with a running buffer containing 20 mM Tris-HCl pH 9.0 and 2 mM CaCl_2_. Proteins were eluted from the column with an increasing NaCl gradient. Fractions containing purified *Mh*PA14 were then pooled and buffer exchanged into Protein Buffer (20 mM Tris-HCl pH 9.0, 150 mM NaCl, and 2 mM CaCl_2_). Protein purity was assessed using SDS-PAGE.

### Size-exclusion Superdex 200 affinity tests

A Superdex 200 10/300 GL column (GE Healthcare) was used to probe the resin-binding ability of *Mh*PA14. Approximately 1 mg of *Mh*PA14 was loaded onto the column and washed with 3 column volumes of Protein Buffer. An increasing gradient of either glucose or EDTA was then implemented, rising from 0 mM– 55 mM glucose or 0 mM– 5 mM EDTA over 45 min.

### Calcium titration and thermal denaturation by Circular Dichroism (CD) spectroscopy

For calcium titrations, *Mh*PA14 (25 μM) was dialysed against Protein Buffer containing no calcium and 5 mM EDTA to ensure calcium removal, before being re-dialyzed into 0.01 mM EDTA. Calcium was then titrated into the protein solution in 0.5 mM increments and mixed thoroughly. For each addition of calcium, eight spectra ranging from 180 nm to 260 nm were taken and averaged using a Chirascan CD Spectrometer (Applied Photophysics). Solutions were maintained at 20°C, throughout.

For thermal denaturation experiments, *Mh*PA14 (25 μM) in either 1 mM CaCl_2_ or 0.01 mM EDTA was subjected to a 5-° incremental increase in temperature from 20°C to 65°C. Multiple scans ranging from 180 nm to 260 nm were taken at each temperature until the spectra stabilized, at which point eight scans were taken and averaged to indicate the equilibrated structural state at that temperature. A refolding procedure, where the temperature was slowly dropped back to 20°C, was also attempted.

All spectra were subjected to three-point smoothing using PROVIEWER software.

### X-ray crystallography and structure solution of *Mh*PA14

The microbatch method was used to screen several crystallization condition suites (Qiagen). *Mh*PA14 at 26 mg/mL was buffer exchanged into 20 mM Tris-HCl pH 9.0 and 10 mM CaCl_2_. The protein solution and varying crystallization conditions were mixed (1 μL:1 μL) in 96-well plates, covered with 100% paraffin oil, and left to incubate at 23° C. The PACT suite yielded many crystals of varying morphologies. These crystals were screened for diffraction using a home source diffractometer (Rigaku MicroMax– 007HF X-ray source coupled to a R-AXIS IV++ detector) in the lab of Dr. John Allingham. Crystals with diffraction around or above 3 Å were either stored or soaked in varying concentrations of sugars, before being analyzed at the Canadian Light Source (CLS) synchrotron. The condition that yielded a glucose-bound structure of *Mh*PA14 contained 0.2 M potassium thiocyanate, 0.1 M Bis-tris Propane pH 7.5 and 20% w/v PEG 3350. The crystal had undergone thirty seconds of soaking in a 30% w/v glucose solution.

Data collected at CLS were indexed and integrated with XDS [38] and scaled with CCP4-Aimless [39]. The structure was solved using molecular replacement with Phenix-Phaser [40]; the sugar-less *Mp*IBP PA14 served as a search model (PDB: **5J6Y**). The initial model was made by Phenix-Autobuild [41] and manually corrected in Coot [42]. Subsequent refinement was done using Phenix-Refine [43].

### Competitive binding assay

Superdex 200 (S200) resin stored in 20% ethanol was washed twice in Protein Buffer using 6600 xg centrifugation. A millilitre of 1 mg/mL *Mh*PA14 fused N-terminally to GFP, named GFP_*Mh*PA14 (S1 Fig), was incubated with 300 μL of equilibrated S200 resin. Following 30 sec of vortexing and 2 min of incubation with slight mixing (nutation), the solution was centrifuged at 6600 xg for 2.5 min and the supernatant discarded. Protein buffer (1 mL) was added to the tube, and the process of mixing followed by centrifugation was repeated. The A280 of the resulting supernatant was taken to account for the minimal amount of non-sugar-related protein disassociation from the resin, and was used as a baseline reading. The supernatant was then returned into its original resin mixture tube with the addition of 1.67 μmoles (3 μL of 555 mM) of saccharide. This was followed by 30 sec of vortexing, 2 min of nutation, and centrifugation at 6600 xg for 2.5 min. After reading the A280 of the supernatant, the above process was repeated for seven tandem saccharide additions of 1.67 μmoles, as well as a final 5-μmole addition. This process is shown as a schematic in S2 Fig.

Data from the dextran-affinity assay were plotted using GraphPad Prism 7.03 and the A280 of non-sugar-related protein disassociation was subtracted. Next, the data were fitted to nonlinear regression of one-site-specific binding, which follows the model *Y/Bmax* = *X*/(*Kd* + *X*), with Bmax as the maximum specific binding and Kd as the equilibrium binding constant.

### Isothermal titration calorimetry (ITC)

Isothermal calorimetric titration (ITC) measurements were performed using a MicroCal VP-ITC calorimeter (Malvern) set at 30°C. *Mh*PA14 in 2 mL of Protein Buffer at a concentration of 400 μM was mixed with 5-μl aliquots from one of four different 8-mM sugar solutions (fucose, glucose, 2-deoxy-D-glucose, and galactose). Sugars were added from the computer-controlled rotating syringe (400 RPM) at 5-min intervals into the *Mh*PA14 solution for a total of 50 injections. The data were analyzed by Origin software Version 5.0 (Malvern).

### Glycan array

Two glycan arrays were probed. The first was done by the Consortium for Functional Glycomics (Harvard Medical School). Version 5.4 of their printed Mammalian glycan array, containing 585 glycans, was incubated with 5 μg/mL, 50 μg/mL, and 200 μg/mL of GFP_*Mh*PA14. The green fluorescence of the fusion protein was used to measure the relative fluorescence units (RFU) of the bound protein. Each glycan was present in six replicates on the array; the highest and lowest value from each set was omitted to avoid false hits, and an average of the remaining four replicates was used.

The second array was screened at the Carbohydrate Microarray Facility (Glycosciences Laboratory, Imperial College). GFP_*Mh*PA14 (50 μg/mL) was exposed to the ‘Fungal, bacterial and plant polysaccharide array set 2’, which contained duplicates of 20 saccharide probes extracted from a variety of organisms. An Alexa Fluora 647-tagged anti-GFP antibody was used for detection, and the duplicates were averaged to give the final RFU values.

## Results

### Recombinant *Mh*PA14 is highly expressed and easily purified

**Fig 2:**
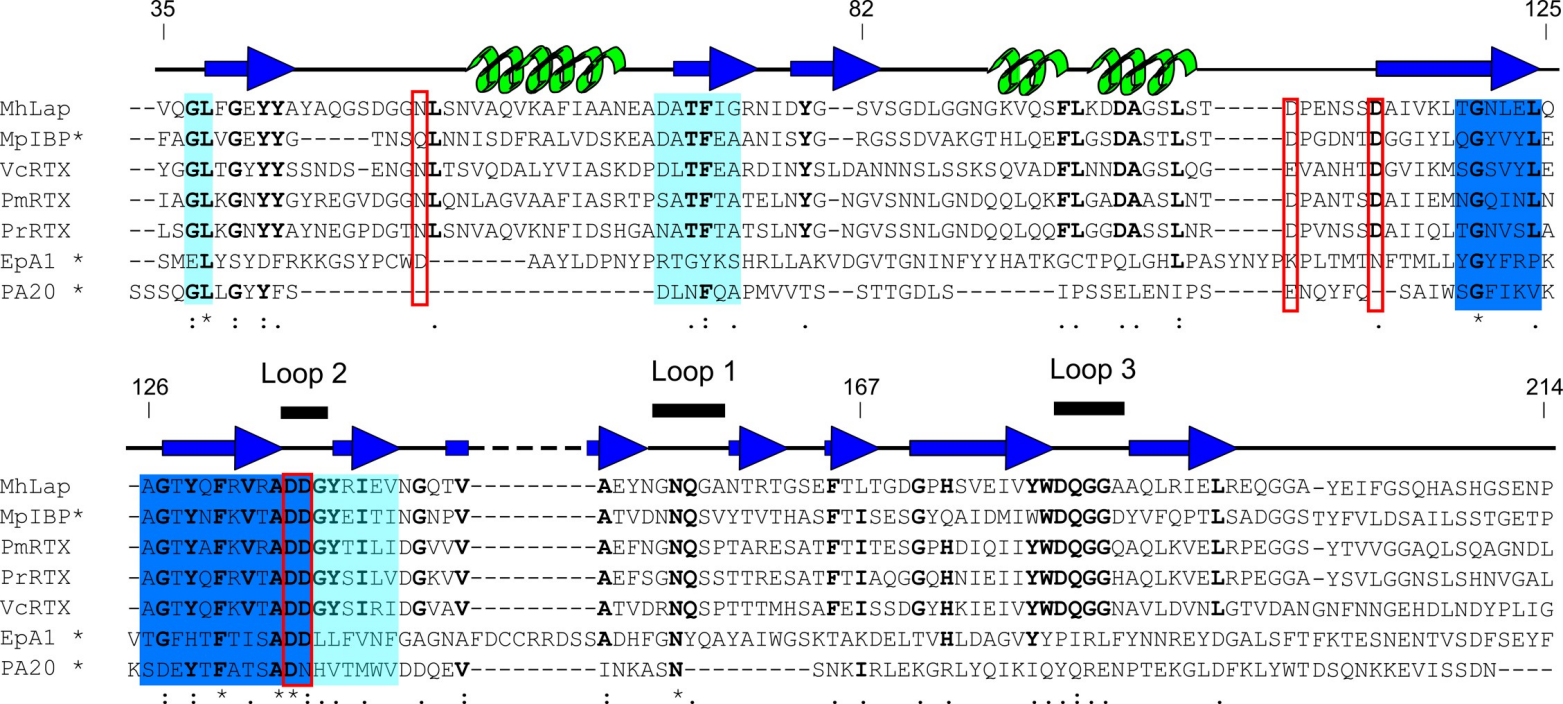
Sequence alignment of PA14 domains. Sequences of PA14 domains from *Marinobacter hydrocarbonoclasticus* (*Mh*Lap), *Marinomonas primoryensis* (*Mp*IBP), *Pseudomonas mendocina* and *resinovorans* (*Pm*RTX and *Pr*RTX, respectively), *Vibrio cholerae* (*Vc*RTX), *Candida glabrata* (EpA1), and *Bacillus anthracis* (PA20) were aligned using the MergeAlign software [45]. Sequences with alignment scores above 60% are coloured dark blue, while sequences with alignment scores between 30 and 40% are shown in light blue. Proposed Ca^2+^-binding residues are boxed with red squares. Residue conservation is indicated as follows: * = 100%,: = ~80%,. = ~70%. The conserved residues are bolded. Residue numbers for the *Mh*PA14 construct are given. Secondary structure taken from the solved structure of *Mp*IBP PA14 domain is shown above the alignment, with beta-strands coloured dark blue, alpha-helices coloured green, and loops coloured black. Bold lines sit above the sequence for three loops that make up the supposed sugar-binding site of the bacterial PA14 domains. Note: sequences with previously solved protein structures are marked with an asterisk.

Full-length *Mh*Lap is a 3443-residue protein (NCBI: **WP_014422746**) that follows the conserved domain architecture of most RTX adhesins (Fig 1). To extract the PA14 domain for recombinant expression, the Pfam database was used to locate the general N- and C-terminal ends of the domain. This was followed by a series of BLAST searches, multiple sequence alignments, and Phyre2 modelling sessions to designate specific start and stop sequences for the recombinant *Mh*PA14 construct (S1 Fig). Alignment of several RTX adhesin PA14 domains from different *Gammaproteobacteria* species (Fig 2) shows that *Mh*PA14 shares ~50% sequence identity with domains from the two *Pseudomonas* species RTX adhesins, and ~40% to the adhesins of both *Vibrio cholerae* and *M*. *primoryensis*, which agrees with the phylogenetic relationship of the bacteria’s genera [44]. The residues that make up definitive calcium-binding sites in *Mp*IBP [11] are highly conserved amongst the other RTX adhesins, as seen by the red boxed and bolded residues in Fig 2. However, when more-distantly-related proteins like Epa1 and PA are added to the alignment, only small areas of overlap remain, specifically the sequence surrounding the D-cis-D motif, shown in dark blue (Fig 2).

**Fig 3:**
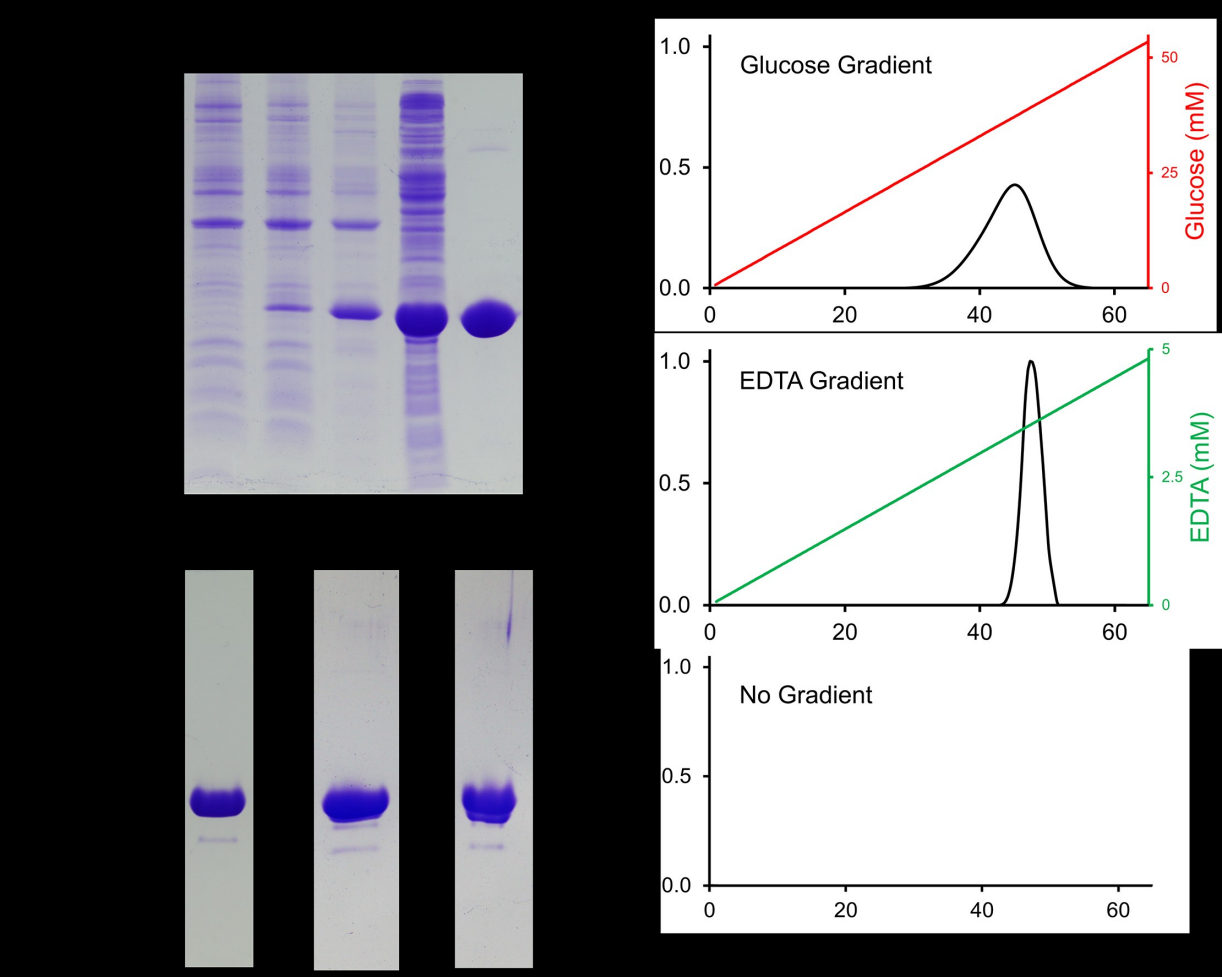
*Mh*PA14 purification and affinity for Superdex 200 size-exclusion resin. A) SDS-PAGE tracking of *Mh*PA14 purification through induction, extraction, and nickel-affinity chromatography. Lane 1 = pre-induction, Lane 2 = post-induction, Lane 3 = lysate insoluble fraction, Lane 4 = lysate soluble fraction, Lane 5 = elution from nickel-NTA agarose column. B) SDS-PAGE showing secondary purification of *Mh*PA14 following nickel-affinity via anion-exchange chromatography (QSeph Lane), as well as the pooled fractions following S200-affinity elution with glucose and EDTA, respectively. C) Elution profiles of *Mh*PA14 interacting with a Superdex 200 size-exclusion column under varying gradient conditions. The absolute absorbance at 280 nm (primary y-axis) was measured over 65 mL of buffer flow, during which an glucose gradient (top, red), an EDTA gradient (middle, green), or no gradient (bottom) was run.

The codon-optimized version of the 22.5-kDa *Mh*PA14 was well-expressed, and preferentially partitioned into the soluble fraction upon cell lysis (Fig 3A). A two-step purification process of nickel-affinity chromatography, followed by Q-Sepharose anion-exchange chromatography, produced a relatively pure protein devoid of major contaminants (Fig 3A and 3B) that migrated at the expected position for its molecular weight. The yield was ~90 mg of protein per 1 L of *E*. *coli* culture. However, *Mh*PA14 eluted from the anion-exchange column over five 3-mL fractions as a doublet peak. When run on SDS-PAGE, both doublet peaks contained indistinguishable *Mh*PA14 bands, along with a miniscule amount of contaminant at ~18 kDa (Fig 3B).

When size-exclusion chromatography was attempted, *Mh*PA14 failed to elute from the column, even after several column volumes of buffer were passed through. An affinity for a component of the polysaccharide-based size-exclusion resin was suspected due to the proposed sugar-binding nature of the PA14 domain. The glucose polymer, dextran, was singled out as the most likely ligand, as the protein was retained by both Superdex (dextran and agarose) and Sephadex (dextran), but not Sepharose (agarose). To test this suspicion, a known quantity of purified *Mh*PA14 was bound to a Superdex S200 column and subjected to an increasing glucose gradient. The protein began eluting at a glucose concentration of 25 mM and did so over the course of 20 mL, producing a broad, dispersed peak of *Mh*PA14 (Fig 3C, top). SDS-PAGE was used to confirm that the pooled fractions that eluted off the S200 were, in fact, *Mh*PA14, comparable in size and purity to the Q-Sepharose-purified protein (Fig 3B).

Since the alignment in Fig 2 demonstrated that the D-cis-D motif–which uses calcium for sugar recognition–was conserved in *Mh*PA14, the divalent ion-chelator EDTA was also used as a potential eluting agent (Fig 3C, middle). Indeed, 3.5 mM of EDTA led to the elution of *Mh*PA14 as a sharper peak over < 10 mL. Both elution methods were compared to a control run where no gradient was added (Fig 3C, bottom), which failed to elute any protein. Interestingly, both sub-peaks from the anion exchange-purified doublet peak were able to bind to the resin, indicating that these peaks likely contain different conformers of *Mh*PA14, each retaining a common functionality.

### *Mh*PA14 requires calcium for proper folding and stability

**Fig 4:**
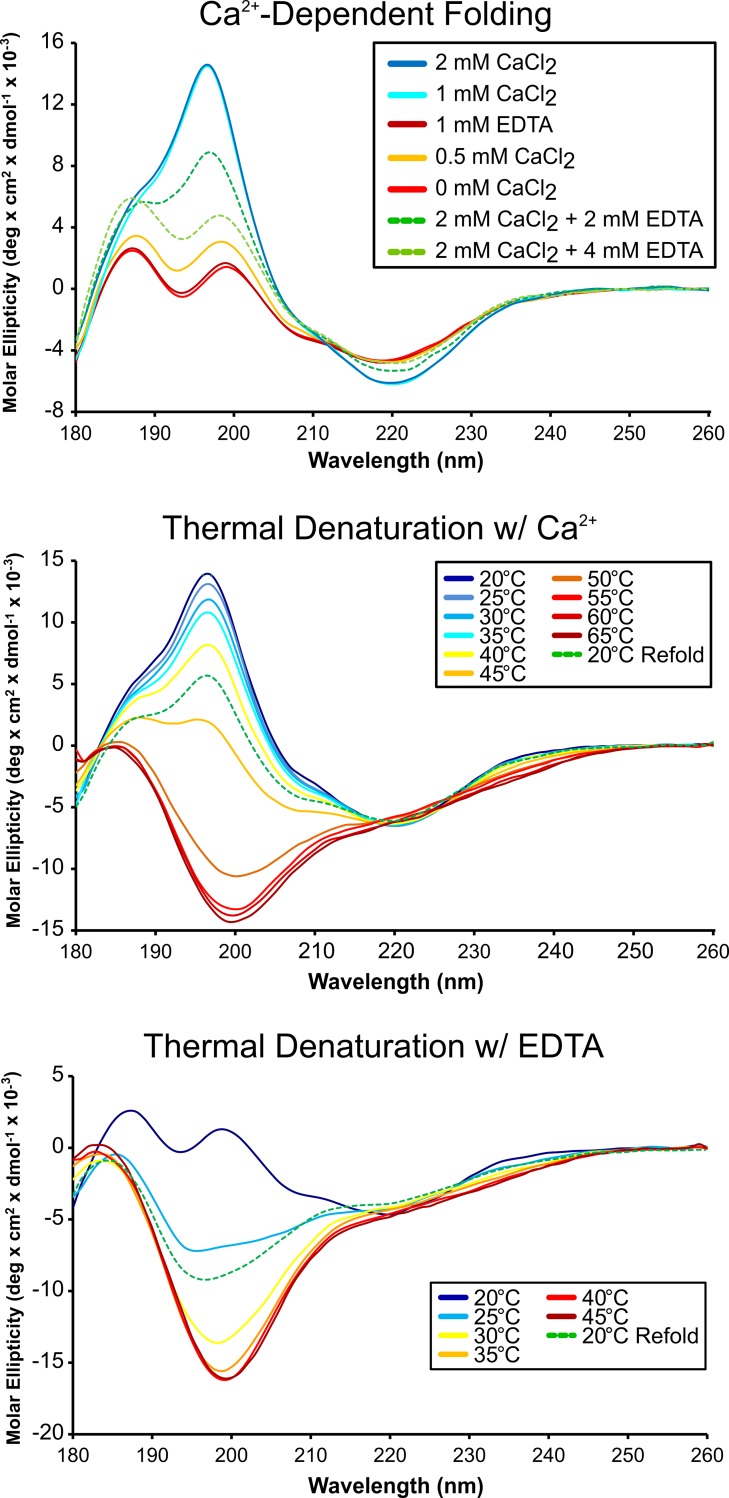
Circular dichroism spectra assaying calcium dependence and thermal denaturation of *Mh*PA14 structure. Overlaid CD spectra of *Mh*PA14 (25 μM) during a Ca^2+^ titration (top), and increasing temperature in the presence of Ca^2+^ (middle) or EDTA (bottom).

The elution of *Mh*PA14 from the dextran-based resin via EDTA made it clear that a divalent cation–presumably calcium–was important for the domain’s sugar affinity. However, it was unclear if this was simply due to the D-cis-D calcium-binding motif previously mentioned, or if there was also a calcium requirement of *Mh*PA14 for proper folding, as there is in previously studied domains taken from RTX adhesins [21,46]. Circular dichroism (CD) spectroscopy was used to assay the effect of calcium on the secondary structure of the domain (Fig 4, top). Purified *Mh*PA14 was dialysed against excess EDTA to remove Ca^2+^, and then transferred to a buffer with minimal (0.01 mM) EDTA in which to titrate CaCl_2_. The CD spectra of *Mh*PA14 in both 1 mM and minimal EDTA solutions took on the same shape, composed of two peaks at 187 nm and 199 nm and a trough at ~220 nm. Titration of CaCl_2_ into the sample with minimal EDTA caused a drastic change in the spectrum. Following the addition of 1 mM CaCl_2_, the two peaks coalesced into one maximum near 195 nm, while the minimum at ~ 220 nm was retained. Such spectra are characteristic of β-sheet dominated structures [47]. This spectral shape was maintained upon further additions of CaCl_2_, indicating that the *Mh*PA14 calcium-interaction was saturated by 1 mM of titrant. The addition of EDTA back into the sample lead to an incremental return to the former shape.

Thermal denaturation studies were undertaken in both the presence (Fig 4, middle) and absence (Fig 4, bottom) of Ca^2+^ to test the stability of the structures seen at the beginning and end of the calcium titration. In the presence of 1 mM CaCl_2_, the PA14 domain maintained its spectral shape until 45°C, at which point a shift towards a characteristic “unfolded” spectrum was observed. By 55°C, *Mh*PA14 appeared entirely unfolded. In contrast, the dual-peak shape of the protein’s CD spectrum in EDTA began to transition after only a 5-°C temperature increase, and the protein was almost completely unfolded at 30°C. In both conditions, *Mh*PA14 was unable to fully regain its former structure upon returning to 20°C, though the refolding was more successful in the presence of Ca^2+^ (Fig 4).

### *Mh*PA14 crystal structure reveals three bound Ca^2+^, and one glucose

Purified *Mh*PA14 was crystallized under several conditions–all of them sugar-free–with the best condition producing thick, prismatic crystals that diffracted to ~ 1.2 Å. Crystal soaking experiments were conducted using readily available mono- and disaccharides. While many crystals remained devoid of obvious density in which to place a bound sugar, a glucose-bound dataset was eventually collected and solved using molecular replacement with the *Mp*IBP PA14 domain as a template (Table 2).

**Table 2:** Crystallography collection and refinement statistics for *Mh*PA14.

**Wavelength**	0.9793377
**Resolution range**	39.76–1.198 (1.241–1.198)
**Space group**	P 2 21 21
**Unit cell**	32.575 61.309 79.519 90 90 90
**Total reflections**	487315 (45409)
**Unique reflections**	50698 (4815)
**Multiplicity**	9.6 (9.4)
**Completeness (%)**	99.59 (95.93)
**Mean I/sigma(I)**	27.60 (16.56)
**Wilson B-factor**	7.3
**R-merge**	0.06239 (0.1065)
**R-meas**	0.06587 (0.1127)
**R-pim**	0.02087 (0.03637)
**CC1/2**	0.998 (0.995)
CC*	1 (0.999)
**Reflections used in refinement**	50697 (4814)
**Reflections used for R-free**	2442 (254)
**R-work/R-free**	0.140/0.159
**CC(work)**	0.852 (0.746)
**CC(free)**	0.825 (0.788)
**RMS(bonds)**	0.011
**RMS(angles)**	1.05
**Ramachandran favored (%)**	97.14
**Ramachandran allowed (%)**	2.29
**Ramachandran outliers (%)**	0.57
**Rotamer outliers (%)**	0
**Clashscore**	1.16
**Average B-factor**	9.65
** macromolecules**	8.4
** ligands**	7.97
** solvent**	17.13

**Fig 5:**
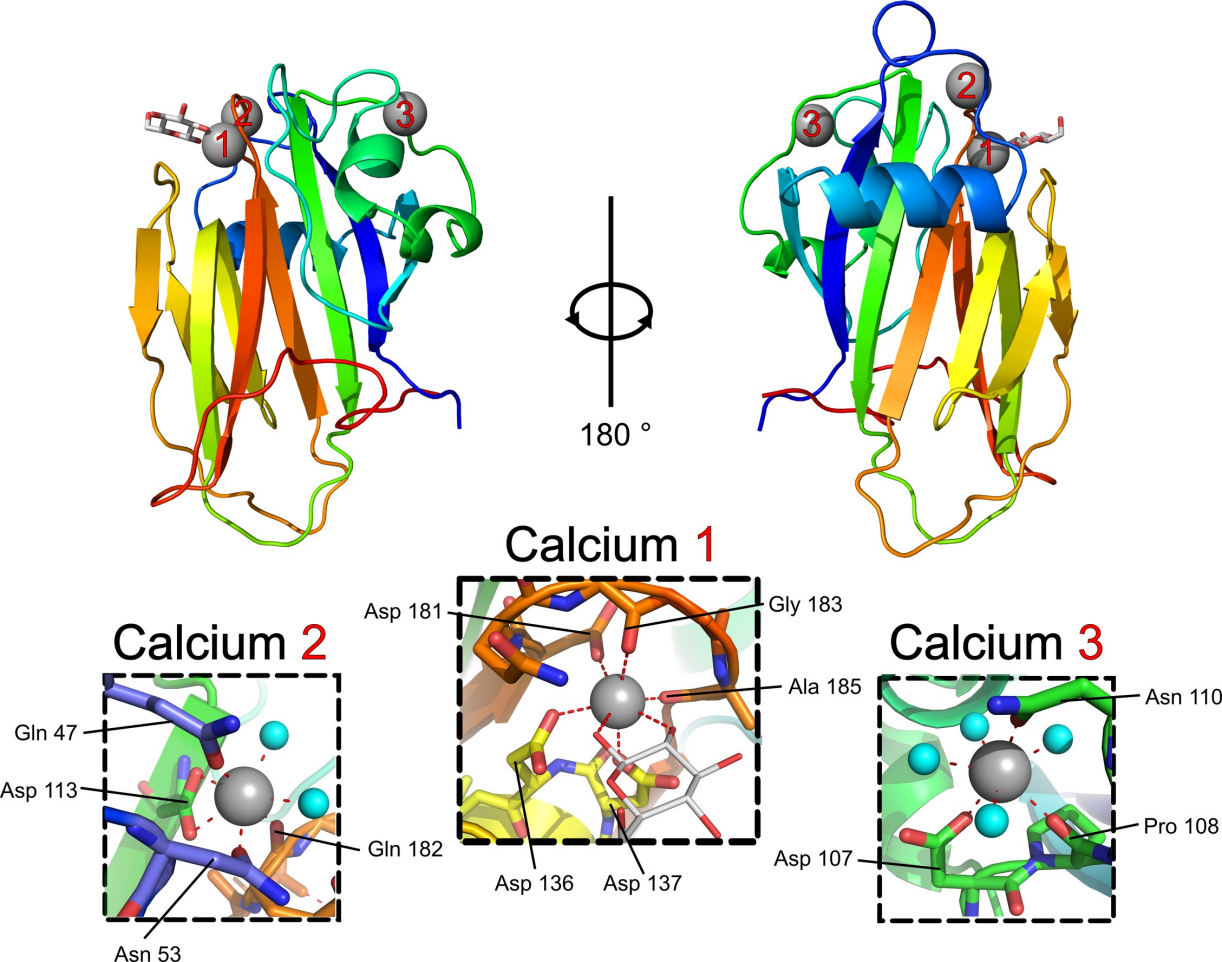
*Mh*PA14 structure with glucose bound. The structure for *Mh*PA14 presented as a cartoon diagram from two viewpoints, 180° rotated. The protein is coloured by progression of primary structure from the N terminus in blue, to the C terminus in red. Calcium ions are shown as grey spheres, coordinated water molecules are shown as smaller cyan spheres, and the glucose molecule is shown as a stick structure with white for carbon and red for oxygen. Inset panels detailing the coordination for each calcium ion are shown, with red dashed lines indicating coordinate bonds between the calcium and the labelled residue or water molecule.

*Mh*PA14 takes on a beta-sandwich-type fold, comprised primarily of two anti-parallel beta-sheets held together by a hydrophobic core (Fig 5). Two small alpha helices contribute to the hydrophobic core, and a third helix lies across the outer surface of the larger beta-sheet. A series of long loops lacking defined secondary structure protrude from the top and bottom of the beta-sheets, as shown in Fig 5. The atoms within the bottom loops have higher B factors compared to the rest of the structure, possibly indicating that these loops are in a relative state of disorder. In contrast, the top loops have similar B factors to the core secondary structure elements, indicating a more ordered region. This is likely a result of the three calcium ions coordinated within these loops (Fig 5).

Calcium 2 and 3 appear to have purely structural roles by forming stabilizing contacts between otherwise disordered portions of the protein. Calcium 2 coordinates residues from two loop regions (using Gln47 and Asn53 from one, and Gln182 from another) and pins them to residue Asp 113, which is part of a beta strand (Fig 5, inset Calcium 2). Meanwhile, calcium 3 is involved in a more local interaction, coordinating three neighbouring residues (Asp107, Pro108 and Asp 110) into a sharp turn (Fig 5, inset Calcium 3). These interactions likely help facilitate the more thermo-stable, beta-strand-rich, calcium-dependent tertiary structure observed in Fig 4.

**Fig 6:**
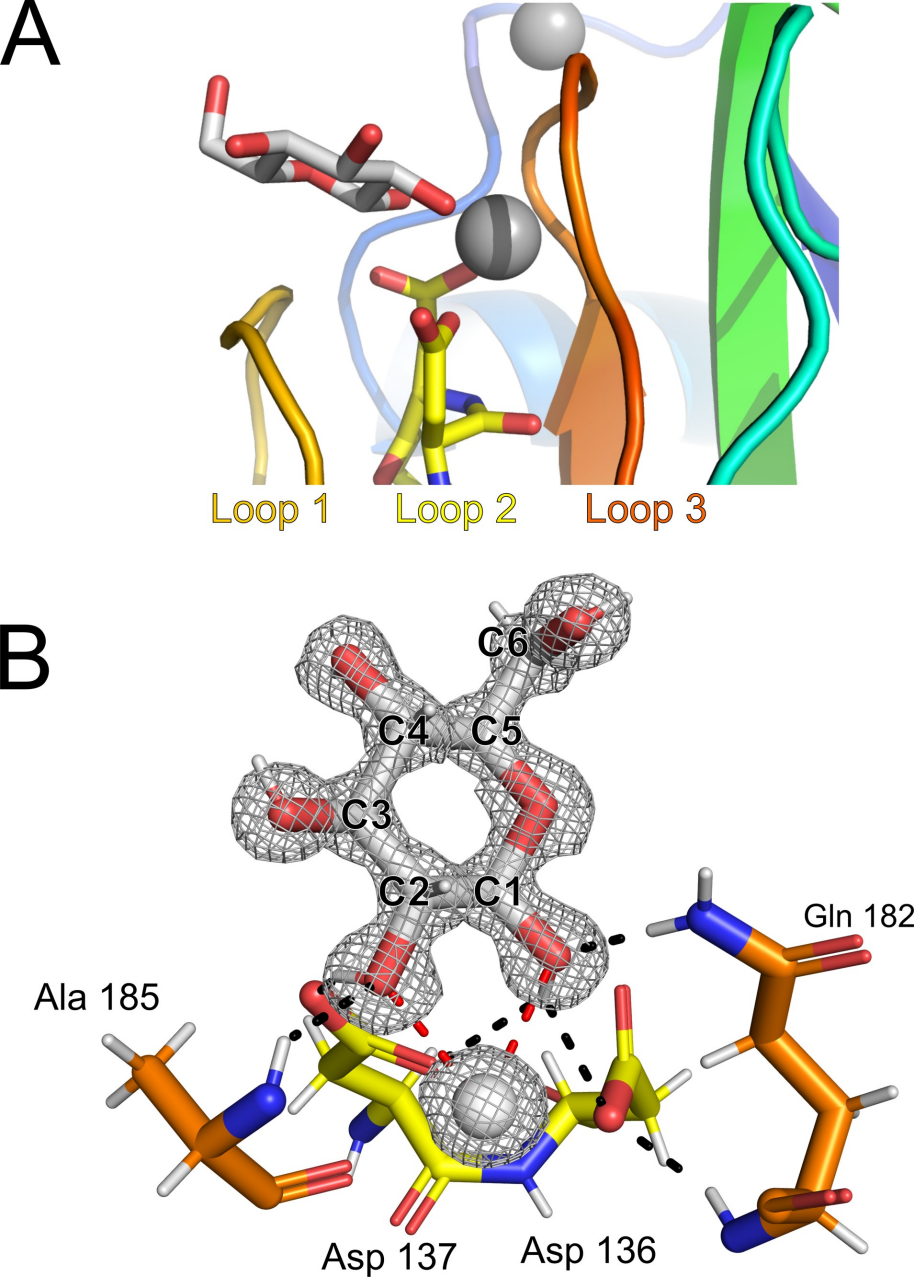
Sugar-binding site of *Mh*PA14. A) Side view of the sugar-binding cleft, produced by loops 1, 2, and 3. Calcium 1 and its coordinated glucose molecule are shown as a grey sphere and white ring, respectively. B) Important residues of the sugar-binding site. Residues are labelled and coloured the same as for Fig 5. Calcium 1 is reduced in size to improve visibility. Red dashed lines are contacts between sugar and calcium, while black dashed lines are between sugar and protein. The 2 F_o_−F_c_ map (σ = 1.5) of the glucose molecule and bound calcium are shown as a grey mesh with carbon atoms of the hexose numbered.

Calcium 1 coordination is also likely to have structural ramifications, pinching two of the top loops together through contacts between the expected D-cis-D motif (Asp136 and 137) in one loop, as well as an additional aspartate and two backbone carbonyl groups from another loop (Asp181, Gly183 and Ala185) (Fig 5, inset Calcium 1). However, calcium 1 –and the three loops that surround it (Fig 6A)–also make up the sugar-binding site, where the structure of *Mh*PA14 reveals a beta-D-glucose molecule to be bound. The sugar is coordinated to calcium 1 through the two vicinal hydroxyl groups attached to the first and second (C1 and C2) carbons in the pyranose ring (Fig 6B). Polar contacts directly between the glucose molecule and the protein are also made via the C1 –C2 hydroxyl pair, with C1 forming contacts with the sidechains of Asp 136, Asp 137 and Gln 182, and C2 connecting to Asp 137 and the backbone of Ala 185 (Fig 6B). The sugar ring also makes several non-polar contacts with the backbone of Loop 1. There is no evidence for an alternative coordination pattern within the distinct electron density (Fig 6B), nor is there any sign of the C1 anomer alpha-glucose.

### *Mh*PA14 crystal structure and sugar orientation compared to other known PA14 domains

**Fig 7:**
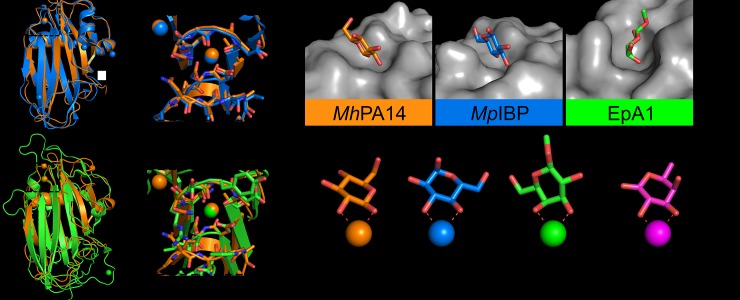
Structural comparison of *Mh*PA14 to other PA14 domains. Structural alignment of *Mh*PA14 (orange) to either A) *Mp*IBP PA14 (blue) or B) EpA1 A domain (green). Close-up views of the sugar-binding site for both alignments are shown in boxes. C) Sugar-binding pockets of these three PA14 structures. Protein topology is coloured grey, sugars are coloured with oxygen in red, nitrogen in blue, and the carbon atoms in the respective colours of their structures shown in A and B. D) The orientation of these same sugars as they coordinate to the calcium ions in their respective structures. E) Orientation of fucose (purple) from LecB.

Structures from other PA14 domains, including *Mp*IBP PA14 (PDB: **5J6Y**) and EpA1 (PDB: **4A3X**) [48], have previously implicated calcium 1 as the prospective cofactor for sugar binding. Indeed, aligning the structure of *Mh*PA14 against both *Mp*IBP PA14 (RMSD = 0.897) and EpA1 (RMSD = 2.372) (Fig 7A and 7B) demonstrated the strong overlap of Loops 1, 2 and 3 that make up the sugar-binding site. As foreshadowed by the amino-acid sequence identities, the PA14 domains of *Mh*Lap and *Mp*IBP are more similar in structure than they are to EpA1, yielding a common ligand-binding site for both structures with an open, flat topology in which their shared ligand–glucose–can reside (Fig 7C). While both structures bind glucose, they do so in different manners, with the glucose in *Mp*IBP interacting with the calcium ion through the C3 and C4 hydroxyl groups, rather than the beta-C1 and C2 seen in *Mh*Lap. That said, both sets of hydroxyls are similarly oriented relative to one another–both being vicinal, trans isomeric, and equatorial–and therefore both appear capable of satisfying the calcium coordination sphere (Fig 7D).

Contrarily, the EpA1 PA14 specifically binds galactose by coordinating its C3 –C4 hydroxyl pair, which are cis isomers, one equatorial and the other axially oriented. This orientation would appear to break the coordination scheme used by the other PA14 domains mentioned thus far. However, the EpA1 sugar-binding region differs greatly from that of *Mh*PA14 and *Mp*IBP, as seen in Fig 7C. The calcium is held in a narrower binding cleft, with large, bulky residues in Loops 1 and 3 restricting the accessibility of the site. The same loops in the other PA14 structures are comprised of residues with small sidechains, predominantly alanine and glycine. Due to the restricted access to the calcium in EpA1, a binding saccharide must be oriented at a different angle than the other two structures, an angle that would not allow trans, equatorial hydroxyl pairs to properly coordinate to the calcium. However, this orientation does allow for the galactose C3 –C4 hydroxyl pair to satisfy the same coordination sphere, as seen in Fig 7D.

### *Mh*PA14 has varying affinity for different sugars, shown by both competitive binding assay and ITC

**Fig 8:**
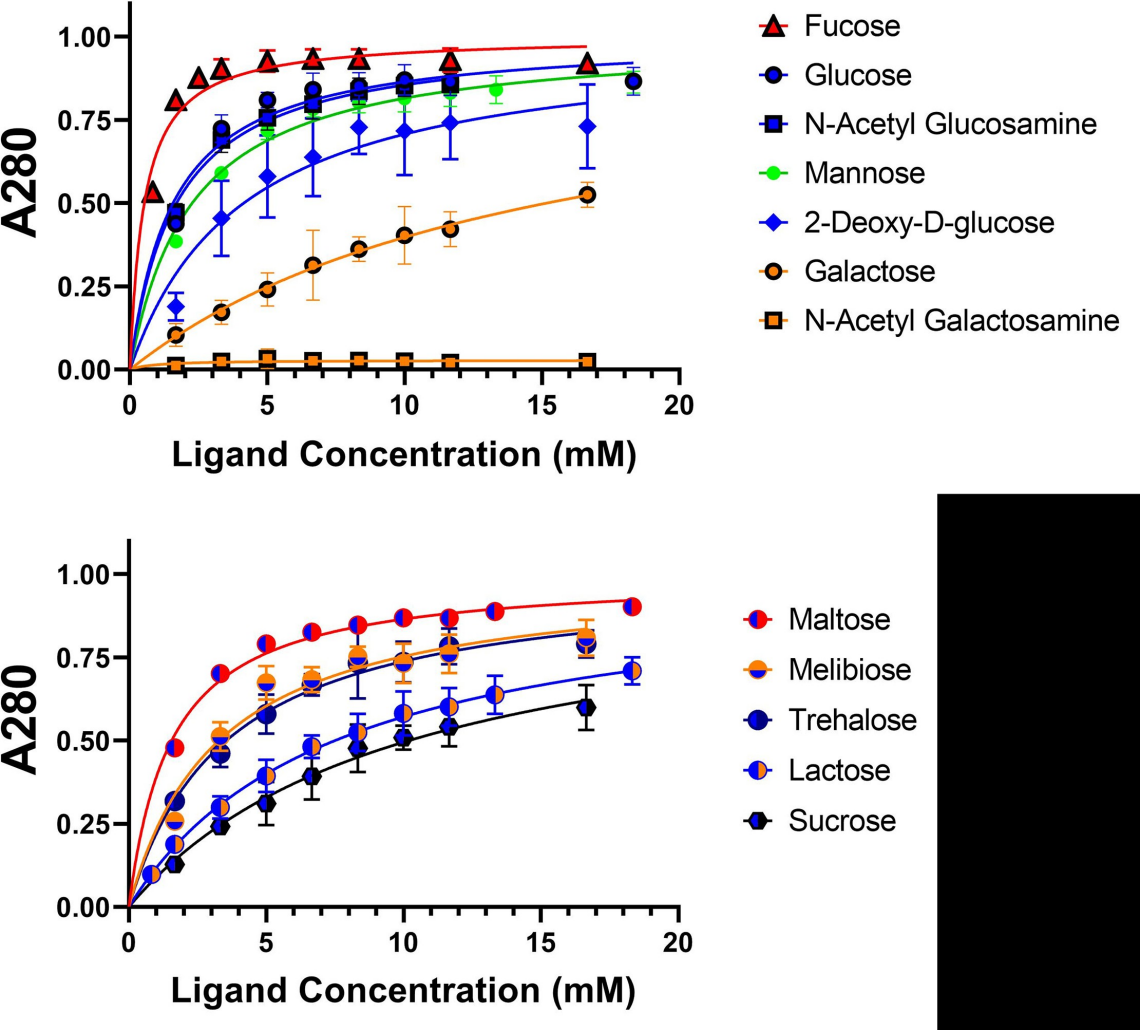
Dextran-based competitive binding assay to determine relative affinity of *Mh*PA14 for sugars. The progressive removal of GFP_*Mh*PA14 from dextran-based resin–using a series of monosaccharide hexoses (top) and disaccharides (bottom)–is shown. The presence of released protein was measured using absorbance at 280 nm, normalized to the maximum released protein value (Bmax). Data points were done in triplicate, with standard deviation shown via error bars. Data were fit with non-linear regression.

Attempts to solve *Mh*PA14 structures with other sugars bounds–whether by soaking or co-crystallization–were fruitless. Therefore, an assay was developed to test the relative affinity of *Mh*PA14 for different sugars. This simple, competitive binding assay was done in 1.5-mL microcentrifuge tubes with *Mh*PA14 bound to Superdex 200 (dextran-agarose) resin and released upon addition of free sugar (S2 Fig). The stronger the binding between *Mh*PA14 and the free sugar (relative to that of the resin), the less sugar it took to completely elute the *Mh*PA14 from the resin. The amount of GFP-tagged *Mh*PA14 released from the resin upon each sugar addition was measured using absorbance at 280 nm, which was plotted against concentration of free sugar to produce semi-quantitative binding curves (Fig 8). A range of sugars, including monosaccharide hexoses and disaccharides, were used for these assays (S3 Fig). The data for each sugar were fitted using non-linear regression to a simple ligand-binding equation assuming a single binding site, which allowed for the calculation of an apparent dissociation constant (K_d_app): a semi-quantitative measure of a sugar’s ability to displace the *Mh*PA14 from the resin (Table 3).

**Table 3:** Apparent dissociation constants for the binding of different sugars to *Mh*PA14, using the competitive binding assay.

	K_d_app (mM)	
**Monosaccharides**		
Fucose	0.52 ± 0.06	
Glucose	1.57 ± 0.22	
N-acetylglucosamine	1.69 ± 0.18	
Mannose	2.26 ± 0.20	
2-deoxy-D-glucose	4.14 ± 1.25	
Galactose	15.16 ± 4.71	
N-acetylgalactosamine	Non-binding	
**Disaccharides**		
Maltose	1.57 ± 0.10	
Melibiose	3.28 ± 0.52	
Trehalose	3.55 ± 0.51	
Lactose	7.50 ± 0.93	
Sucrose	10.19 ± 2.19	

The data from Fig 8 and Table 3 show that the monosaccharides cluster into four groups, in terms of binding affinity. The highest affinity group is populated solely by fucose, also known as 6-deoxy-L-galactose. This sugar has yet to be seen bound to a PA14 domain, though certain C-type lectins–such as LecB (PDB: **1OXC**)–have been shown to bind fucose through calcium coordination via the C2 and C3 hydroxyls (Fig 7E).The second highest grouping contains the hexoses glucose, N-acetylglucosamine, and mannose, all with close to overlapping affinities. The third group holds only the intermediate binder 2-deoxy-D-glucose, with an apparent affinity two-fold lower than fully-oxygenated glucose. The final group consists of the weak binder galactose, and its derivative N-acetylgalactosamine, the latter being unable to bind *Mh*PA14 at all. The disaccharides are more clustered together, with maltose (an α1–4 dimer of glucose) showing the highest affinity, followed closely by both trehalose (α1–1α dimer of glucose) and melibiose (galactose α1–6 glucose). Both lactose (galactose β1–4 glucose) and sucrose (glucose α1-β fructose) show comparably lower affinities.

Due to the many unknown variables at play in this assay (i.e. strength of binding to the dextran-based resin, number of closely spaced binding moieties presented by the resin, the disparity in diffusion between the resin and free sugars) these K_d_app values are not comparable to those attained through more quantitative methodologies, such as isothermal titration calorimetry (ITC) or surface plasmon resonance (SPR). However, they can be compared relative to each other, much like IC50 values, as long as the amounts of resin and protein are kept consistent.

**Fig 9:**
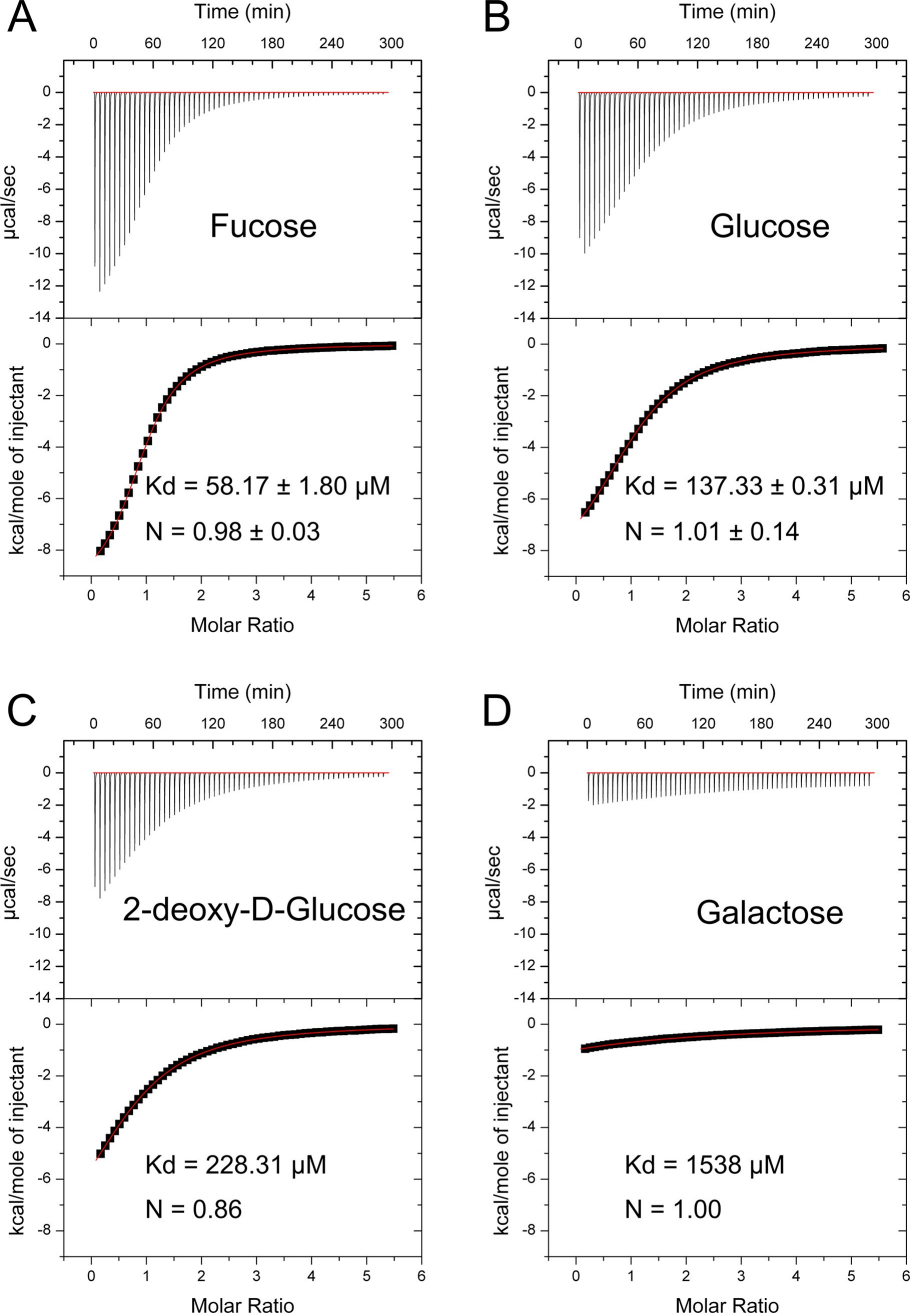
Isothermal titration calorimetry of *Mh*PA14 binding to select sugars. ITC of the interaction between *Mh*PA14 and fucose (A) glucose (B) 2-Deoxy-glucose (C) and galactose (D). A combination of the raw reads over the duration of the titration (top) and the fitted curve (bottom) are shown, along with the calculated K_d_ value for each sugar and the calculated stoichiometry. The fucose and glucose values are averages from triplicate runs.

To ensure that the ordering of sugar affinities is reproducible between the competitive binding assay and more tried-and-true methods, four sugars of apparently different affinities (fucose, glucose, 2-deoxy-glucose, and galactose) were chosen with which to perform ITC (Fig 9). The titration of fucose was the only sugar to show a definitive sigmoidal curve, with the other sugars showing gradual inclines that indicate weak affinity. This makes the inflection point (and therefore the stoichiometry) more difficult to interpret, although it appears to take place at a 1:1 molar ratio of ligand to protein. The fitted curves show a definitive decrease in K_d_ from fucose to glucose to 2-deoxy-glucose to galactose, with values ranging from 58 μM for fucose to above 1 mM for galactose. This supports the order presented by the competitive binding assay, although–as expected–the values themselves are very different. To confirm the quantitative affinity for the strongest binders, fucose and glucose, the ITC experiments were run three times, and the Kd values shown are an average of the triplicate. Interestingly, the glucose 137 μM value is comparable to the dissociation constant between EpA1 and its sugar of choice, galactose (Kd = 115 μM) [48].

### Glycan arrays show *Mh*PA14’s preference for branched glycans with terminal glucose, glucosamine and fucose

**Fig 10:**
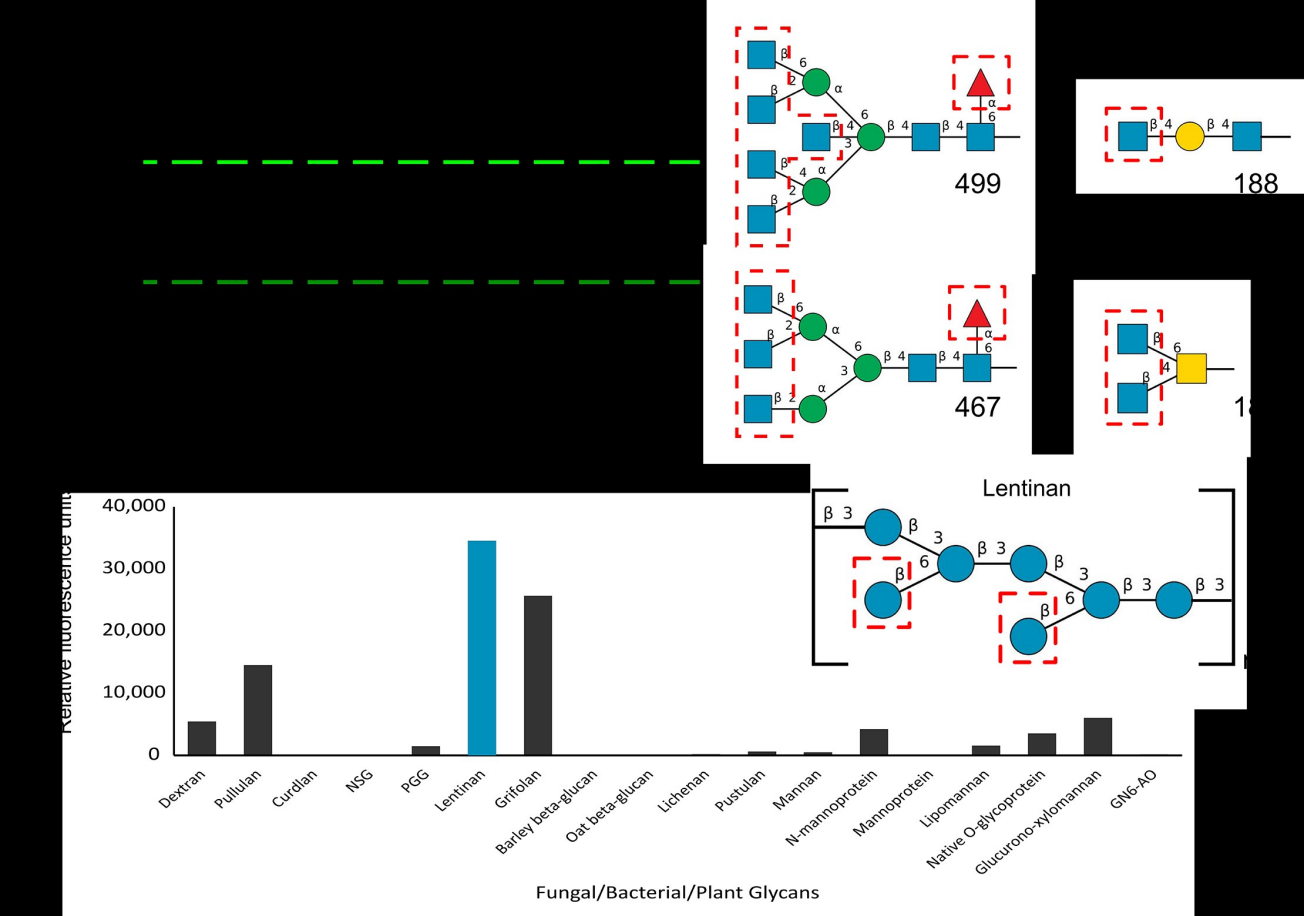
Binding of *Mh*PA14 to Glycan arrays. A) Fluorescent measurements of an array of 585 glycans of varying complexity following incubation with GFP_*Mh*PA14 (200 μg/mL). The fluorescence measured for each glycan is an average of four replicate spots. The four glycan spots that fluoresced above 8000 RFU are labelled, and their structures are presented on the right. Blue squares = N-acetylglucosamine, blue circles = glucose, yellow squares = N-acetylgalactosamine, yellow circles = galactose, green circles = mannose, red triangle = fucose. Terminal sugars proposed to be strong-binders via the competitive assay are outlined in red. B) Fluorescent measurements from a second array, containing eighteen glycans extracted from biological sources following incubation with GFP_*Mh*PA14 (50 ug/mL) and detected through anti-GFP antibody. The fluorescence measured for each glycan is an average of two replicate spots. The highest fluorescing glycan is coloured blue, and its repeating structure is shown on the right using the same colour scheme as in A).

GFP_*Mh*PA14 was incubated with a broad-spectrum glycan array from the Consortium for Functional Glycomics (CFG), in order to expand the assayed sugars beyond simple mono- and disaccharides. In keeping with the 0.1–1 mM affinity seen during ITC, *Mh*PA14 failed to bind to any of the proposed glycans with visible intensity at 5 ug/mL of protein (S4 Fig). Even at 50 ug/mL, only a few potential binders were identified, necessitating a 200 ug/mL incubation to fully capture *Mh*PA14’s glycan binding profile (Fig 10A, left). The four glycans with substantially higher apparent affinity (8000 RFU<) for *Mh*PA14 are all oligomers that contain terminal N-acetylglucosamine moieties: a monosaccharide denoted as a high-affinity binder during the competitive binding assay (Fig 10A, right). In fact, of the twenty glycans that bind with an RFU above 4000, eleven possess terminal N-acetylglucosamines.

The nine glycans in the top twenty binders that do not contain terminal N-acetylglucosamine instead contain a terminal fucose moiety, the supposed strongest binding sugar. Indeed, fucose is also present on glycans 499 and 467, with an apparent role in affinity as the non-fucosylated version of these glycans is bound with less intensity (though still above 4000 RFU). Inversely, the vast majority of glycans that bind with an RFU below 500 lack terminal N-acetylglucosamine or fucose, instead sporting galactose, N-acetylgalactosamine, and neuraminic acid. Unfortunately, this particular array has few glycans containing terminal glucose, though those that are present surprisingly have middling to poor binding.

A more directed array that contains extracted glycans from fungal, bacterial, and plant sources was conducted at the Imperial College Glycosciences Laboratory (Fig 10B). While this array contains significantly fewer glycans to sample, the glycans are explicitly related to biologically-relevant polysaccharides, and the array has various examples of large glucose oligomers missing from the CFG array. Indeed, *Mh*PA14 showed preference for several glucose oligomers, including modest affinities for dextran (an alpha 1–6 oligomer of glucose, and a component of size-exclusion resin), and pullulan (an alpha 1–6 / alpha 1–4 oligomer of glucose). But the strongest affinity was for two fungal beta 1–3 glucans: lentinan and grifolan (chains of beta 1–3 linked glucose molecules, with beta 1–6 branches at varying regularities). The latter, a polysaccharide from *Lentinula edodes* called lentinan, has a predicted structure of two glucose branches every five repeats of the main chain [49]. At a predicted molecular weight of ~ 1, 000, 000 Da [50], that means this seven-glucose unit (Fig 10B, right) could be repeated almost 800 times, leaving 1600 terminal glucose molecules open for interaction. The second-strongest binder, grifolan, is predicted to have a similar structure. Meanwhile, the unbranched beta 1–3 glucan, curdlan [51], has almost no affinity for *Mh*PA14, validating the importance of these terminal sugars for binding.

## Discussion

### *Mh*PA14 affinity for monosaccharides

The crystal structure of *Mh*PA14 revealed a beta-D-glucose bound, despite minimal contacts with the protein itself. The sugar molecule used two equatorial hydroxyl groups to coordinate a calcium ion, namely the hydroxyls attached to beta-C1 and C2 of the glucose. The orientation of these two hydroxyls appears necessary, both to satisfy the calcium ion’s coordination sphere and to partake in several other hydrogen bonds with residues Asp136, Asp137, Gln182, and Ala 185. Comparison of *Mh*PA14 and *Mp*IBP’s PA14 show that the C3 –C4 hydroxyl pairs are similarly oriented and can also coordinate to calcium in a comparable manner, while hydroxyl pairs that are not similarly vicinal, trans isomeric, and equatorial are unlikely to be able to satisfy these requirements. For instance, it is likely that the alpha-C1 anomer, formed during the spontaneous cyclization reactions that saccharides like glucose undergo, could not bind to the *Mh*PA14 in this way. Similarly, the C2 –C3 hydroxyls in glucose–while being a pair of tandem, equatorial, trans hydroxyls–are oriented opposite to each other in 3-D space relative to the other pairs and will not be able to satisfy the same coordinate sphere.

The competitive binding assay was used to compare the *Mh*PA14’s affinity for different monosaccharides. Considering glucose was the only sugar that bound to *Mh*PA14 during the crystal soaking experiments, it was not surprising to see that glucose showed one of the strongest affinities for the protein with a K_d_app of 1.57 mM. However, it was surprising to see that N-acetylglucosamine binding rivalled that of glucose (K_d_app = 1.69), despite the C2 hydroxyl being replaced with an acetyl group. In addition, 2-deoxy-D-glucose was able to bind with an affinity only slightly below that of glucose, while galactose (which contains the same equatorial beta-C1 and C2 hydroxyls as glucose) bound rather weakly. This ordering of glucose, 2-deoxy-glucose, and galactose is corroborated by the ITC data, and is therefore not an artifact of the dextran resin-based technique. Additionally, the difference in glucose vs. galactose binding cannot be explained by a difference in the ratio of alpha to beta anomer in solution, as Angyal *et al*. observed consistent ratios for both sugars (alpha 36%, beta 64%) [52].

What most of the strong-binding monosaccharides (glucose, mannose, N-acetylglucosamine) *do* have in common is a pair of trans, vicinal, equatorial hydroxyls on the C3 and C4 carbons. This same orientation is lacking in the weak-binding galactose and N-acetylgalactosamine. Therefore, it is hypothesized that both beta-C1 –C2 and C3 –C4 hydroxyls are capable of binding to calcium 1 in *Mh*PA14, but the C3 –C4 hydroxyl pair is predominantly responsible for binding in solution, while the C1 –C2 pair–though preferred in the artificial environment of a crystal–is less common due to its anomerization. The validity of this hypothesis can be tested by its ability to explain the affinity order seen for the hexose sugars. The 2-D sugar drawings in S3 Fig indicate properly-oriented calcium-binding hydroxyl pairs by circling the hydroxyls and colouring them green. The monosaccharides can be split into four groups: 1) those whose beta-C1 –C2 and C3 –C4 hydroxyl pairs are both properly oriented (glucose, mannose); 2) those where only the C3 –C4 hydroxyl pair is properly oriented or available (N-acetylglucosamine, 2-deoxy-glucose); 3) those where only the beta-C1 –C2 hydroxyl pair is properly oriented (galactose); and 4) those that lack both properly oriented pairs (N-acetylgalactosamine). The competitive binding assay shows that all hexoses that fall into the first two categories are strong binders, indicating the importance of the C3 –C4 pair, to the apparent irrelevance of the C1 –C2 pair. However, while the last group comprised solely of N-acetylgalactosamine is practically unable to bind *Mh*PA14, the penultimate group still manages modest binding, which is likely due to the C1 –C2 pair.

The sugar with the most divergent structure, and therefore more difficult to explain, also happens to be the strongest binding ligand recorded here: fucose. Due to the L-conformation of fucose, its hydroxyls are differently oriented relative to the other hexose sugars. Because of this, the C2 and C3 hydroxyls are actually in the correct orientation for calcium coordination, as seen in Fig 7E. These same hydroxyls coordinate calcium in the C-type lectin, LecB, though this structure actually uses a dual calcium motif to further coordinate the sugar to the protein [53], possibly explaining LecB’s enhanced affinity for fucose (Kd = 58 μM) relative to *Mh*PA14 [54]. The improved binding of fucose relative to glucose is likely a result of the C4 axial hydroxyl; manual docking of fucose into the ligand-binding site of *Mh*PA14 shows how this oxygen could form favourable hydrogen bonds with Gln 182 and Asn 154.

### *Mh*PA14 affinity for di- and oligosaccharides

Both EpA1 and the homologous Flocculin 5 (Flo5) from *Saccharomyces cerevisiae* show preferential binding to disaccharides over monosaccharides, due to extra contacts with the second sugar moiety [26,55]. However, none of the disaccharides tested against *Mh*PA14 in the competition-based sugar-binding assay had higher affinity to the protein than glucose, with the highest–maltose (an alpha 1–4 connected dimer of glucose)–sharing the same affinity as glucose. It is likely that all other disaccharides bind through their glucose-based hydroxyl pairs but are hampered in binding by steric interference between the second sugar component and the protein.

The CFG glycan array results supports this reading of the competitive binding assay. Terminal N-acetylglucosamine and/or fucose moieties are present in all the top binders, with no strong preference for a single linkage type that attaches these terminal sugars to the glycan. Indeed, the top four binders contain combinations of beta 1–4, beta 1–2, and beta 1–6 linkages to galactose or mannose secondary sugars. That said, certain linkage types do appear to be detrimental to proper binding. For instance, an exact copy of glycan 188, but with an alpha 1–4 linkage between the terminal N-acetylglucosamine and the inner galactose instead of the beta 1–4 linkage, leads to abysmal binding. Once again, the implication is that steric hindrance between the secondary sugars and the structure can hamper binding to the terminal sugars of interest.

It is possible that *Mh*PA14 makes additional contacts to oligosaccharides not available during these experiments. However, unlike EpA1, which has a more cluttered topology in its sugar-binding region, or the flocculin proteins, which contain an additional subdomain for contacting the second sugar, the sugar-binding regions of *Mh*PA14 and *Mp*IBP are far more open and possibly unable to facilitate additional favourable contacts. It appears likely that the *Mh*PA14 relies mostly–if not entirely–on contacts with a single terminal sugar.

### What is the function of *Mh*PA14 in the long adhesion protein?

The domain architecture of *Mh*Lap places the PA14 domain in an optimal position for interaction with the extracellular environment. In such a role, the adhesin’s sugar binding could facilitate contacts between cells in the *Marinobacter* biofilm through adhering directly to membrane-associated glycans–as occurs in yeast flocculation [55]–or perhaps through communal binding to the many secreted extracellular polysaccharides known to be vital for many bacterial biofilms [56,57]. As an alternative/complementary function, such sugar-binding domains can also forge connections between species, bringing multiple biological skillsets together in symbiotic communities. Such was the case for the previously studied PA14 from *Mp*IBP, which connected its bacterial host to phototrophic diatoms to improve the availability of oxygen[11]. Indeed, examples of *Marinobacter* species forming consortia with different diatom species have been reported and characterized [58–62].

Identifying the ligand(s) that *Mh*PA14 can bind in its environment is not an easy task, though the binding data and structure support the notion that a single sugar moiety can dictate the recognition and binding of the protein to its ligands. Such a broad system for ligand determination is reminiscent of the C-type lectin domains, which bind oligosaccharides using a calcium-based interaction with terminal sugars [63]. As such, any glycans that contain terminal C3 –C4 hydroxyl pairs–while avoiding major steric clashes through the second sugar moiety–could be potential ligands for *Mh*PA14. It, then, stands to reason that highly-branched sugars would serve as better-binding ligands, providing more termini with which to interact. The glycan arrays provide evidence for this preference, as most of the top-binding glycans in the CFG array contain multiple terminal sugars with open C3 –C4 hydroxyl pairs, as do the beta glucans lentinan and grifolan in the Imperial College glycan array. Indeed, the moderate binding affinity of the *Mh*PA14 may play a part in ligand specificity, as only ligands that provide many potential binding sites (such as the dextran-based resins) for several *Mh*Lap adhesins to bind to will properly facilitate a cell-to-glycan interaction via this multi-valent effect. Another possibility that cannot be ruled out is that additional adhesion domains in the *Mh*Lap adhesin contribute to ligand recognition.

## Conclusions

The PA14 domain found at the distal end of the RTX adhesin from *M*. *hydrocarbonoclasticus* is a confirmed sugar-binding domain that uses a coordinated calcium ion to preferentially bind hexose sugars like fucose, glucose, mannose and N-acetylglucosamine. The *Mh*PA14’s natural affinity for dextran-based size-exclusion resin allowed for the design and development of a competitive binding assay, where *Mh*PA14 was competed off the resin through titration of free sugars. This methodology is a quick and cost-effective way to assay many sugars for relative binding affinity and could be a useful tool for studying the many PA14 domains that reside in RTX adhesins, or lectins in general.

## Supporting information

S1 Fig Amino-acid sequence of MhPA14, and GFP_MhPA14 fusion protein. Sequences for the two constructs are shown as combinations of *Mh*PA14 (yellow), GFP (green), and additional vector and linker sequences (grey).
(PDF)

S2 Fig Dextran-affinity assay schematic. A) Overview of the dextran-affinity assay, showing the process from resin equilibration to repetitive addition of sugars. B) Elution of GFP_*Mh*PA14 from S200 beads in 1.5-mL tubes using glucose.
(PDF)

S3 Fig Two-dimensional representations of the saccharides used in the competitive binding assay. Glucose moieties are coloured red, galactose moieties are coloured blue, and fucose moieties are colored magenta. Trans equatorial hydroxyls are coloured green.
(PDF)

S4 Fig CFG glycan array at multiple concentrations of protein. *Mh*PA14 was incubated with the CFG glycan array at three different protein concentrations. The relative fluorescence of each glycan is an average of four replicate spots.
(PDF)

## Abbreviations

*Mh*Lap: *Marinobacter hydrocarbonoclasticus* Long Adhesion Protein
PA14: Protective Antigen 14
RTX: Repeats In Toxin
