# Correction: Structure and functional analysis of a bacterial adhesin sugar-binding domain

**DOI:** 10.1371/journal.pone.0221101

**Published:** 2019-08-08

**Authors:** Tyler D. R. Vance, Shuaiqi Guo, Shayan Assaie-Ardakany, Brigid Conroy, Peter L. Davies

As a result of the typesetting process, Figs 3,7,8 and 10 do not appear correctly. Please see the correct Figs 3,7,8 and 10 here.

**Fig 3:**
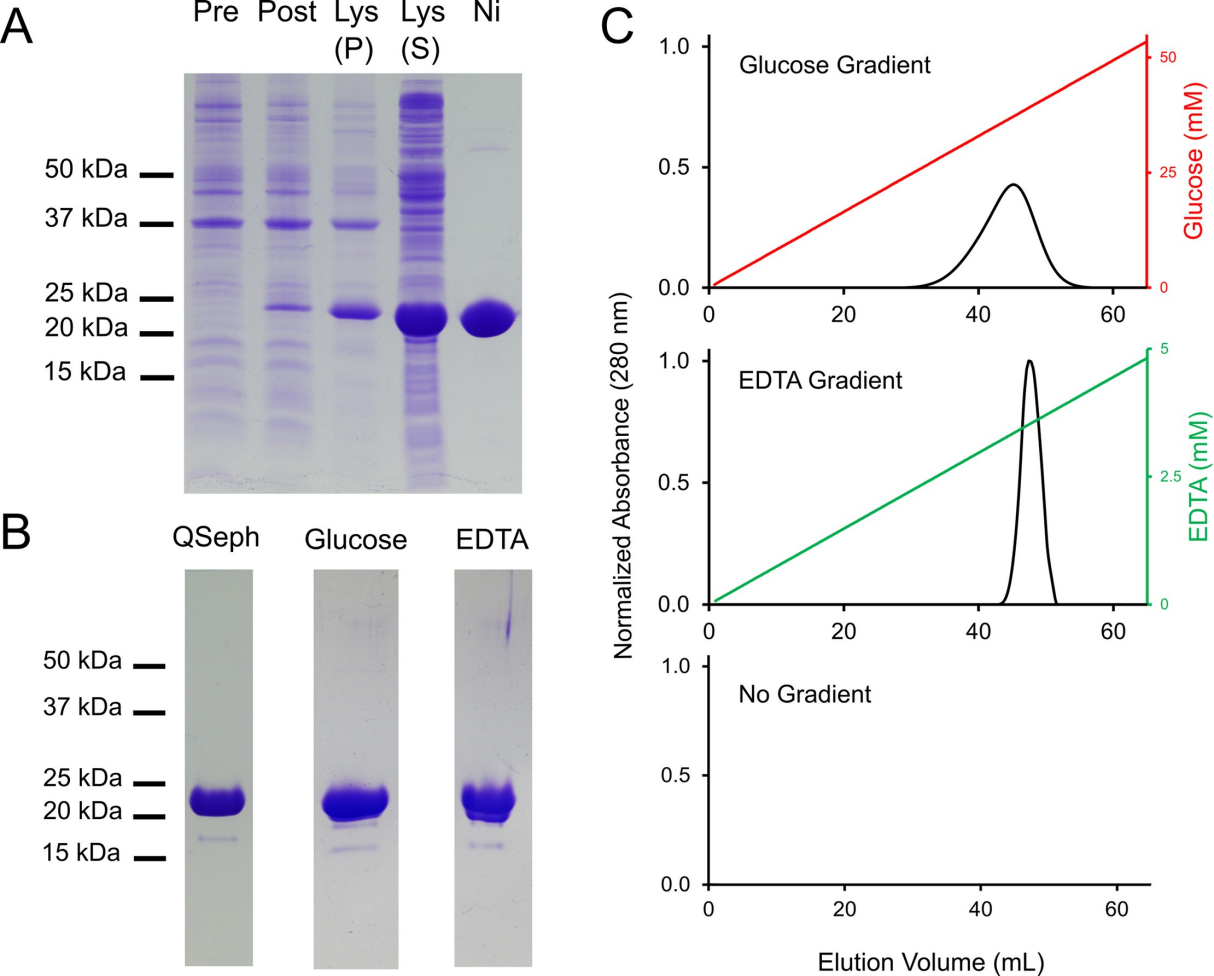
*Mh*PA14 purification and affinity for Superdex 200 size-exclusion resin. A) SDS-PAGE tracking of *Mh*PA14 purification through induction, extraction, and nickel-affinity chromatography. Lane 1 = pre-induction, Lane 2 = post-induction, Lane 3 = lysate insoluble fraction, Lane 4 = lysate soluble fraction, Lane 5 = elution from nickel-NTA agarose column. B) SDS-PAGE showing secondary purification of *Mh*PA14 following nickel-affinity via anion-exchange chromatography (QSeph Lane), as well as the pooled fractions following S200-affinity elution with glucose and EDTA, respectively. C) Elution profiles of *Mh*PA14 interacting with a Superdex 200 size-exclusion column under varying gradient conditions. The absolute absorbance at 280 nm (primary y-axis) was measured over 65 mL of buffer flow, during which an glucose gradient (top, red), an EDTA gradient (middle, green), or no gradient (bottom) was run.

**Fig 7:**
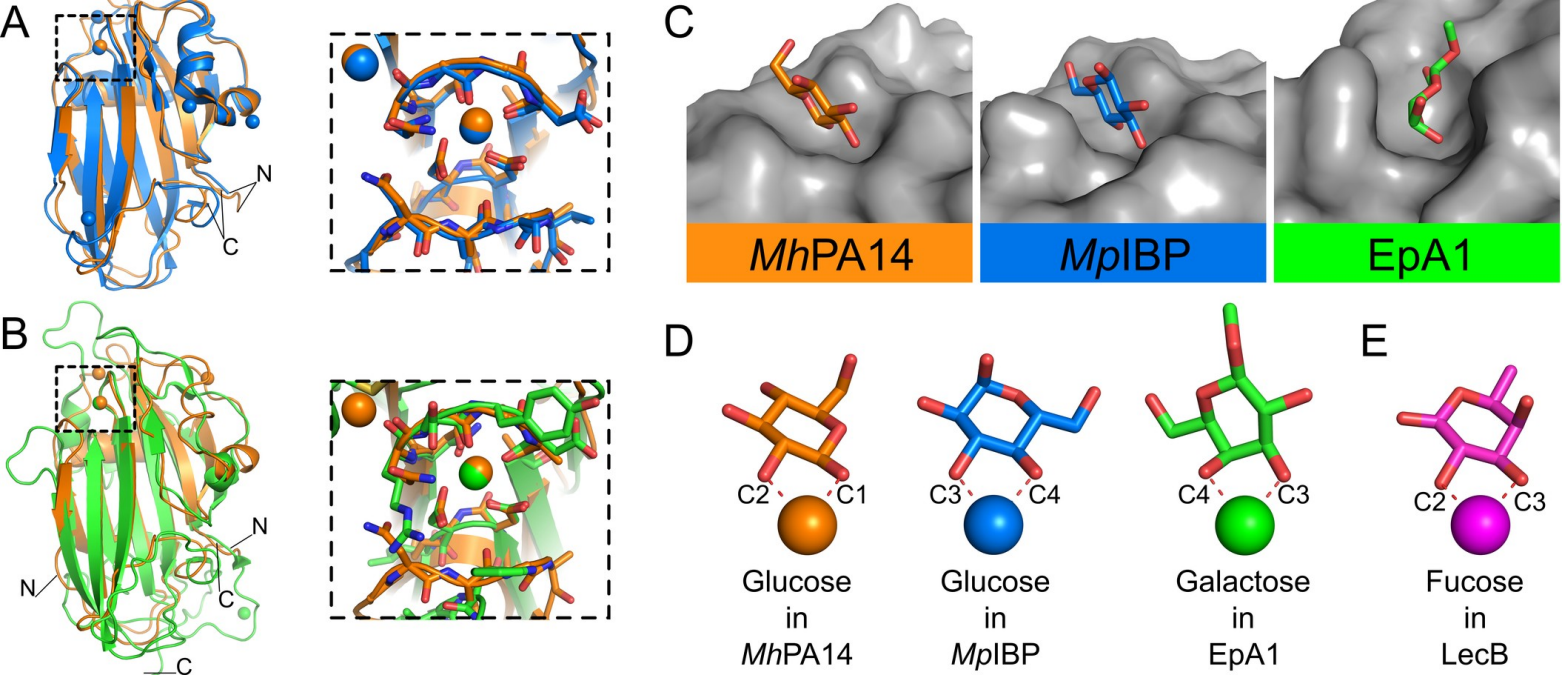
Structural comparison of *Mh*PA14 to other PA14 domains. Structural alignment of *Mh*PA14 (orange) to either A) *Mp*IBP PA14 (blue) or B) EpA1 A domain (green). Close-up views of the sugar-binding site for both alignments are shown in boxes. C) Sugar-binding pockets of these three PA14 structures. Protein topology is coloured grey, sugars are coloured with oxygen in red, nitrogen in blue, and the carbon atoms in the respective colours of their structures shown in A and B. D) The orientation of these same sugars as they coordinate to the calcium ions in their respective structures. E) Orientation of fucose (purple) from LecB.

**Fig 8:**
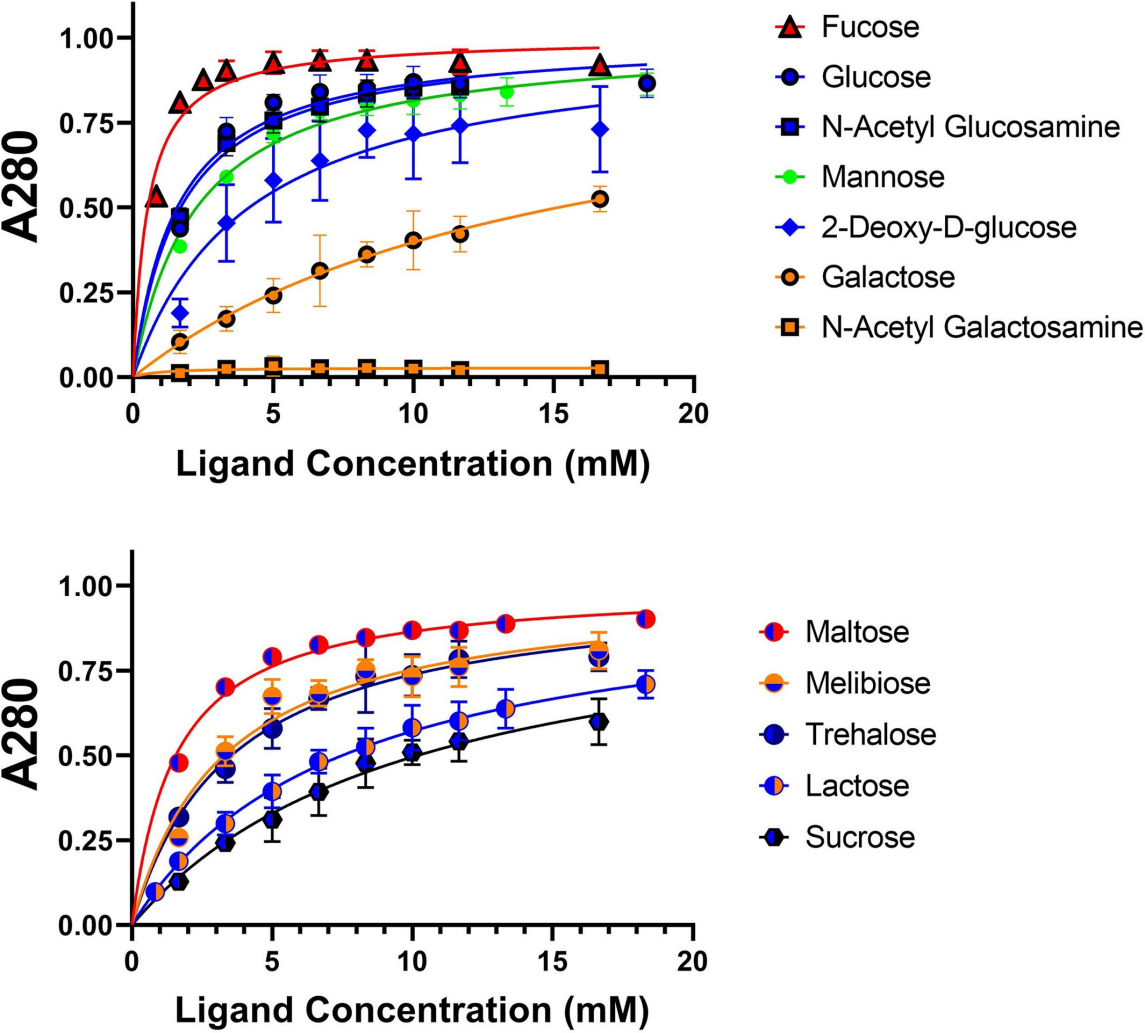
Dextran-based competitive binding assay to determine relative affinity of *Mh*PA14 for sugars. The progressive removal of GFP_*Mh*PA14 from dextran-based resin–using a series of monosaccharide hexoses (top) and disaccharides (bottom)–is shown. The presence of released protein was measured using absorbance at 280 nm, normalized to the maximum released protein value (Bmax). Data points were done in triplicate, with standard deviation shown via error bars. Data were fit with non-linear regression.

**Fig 10:**
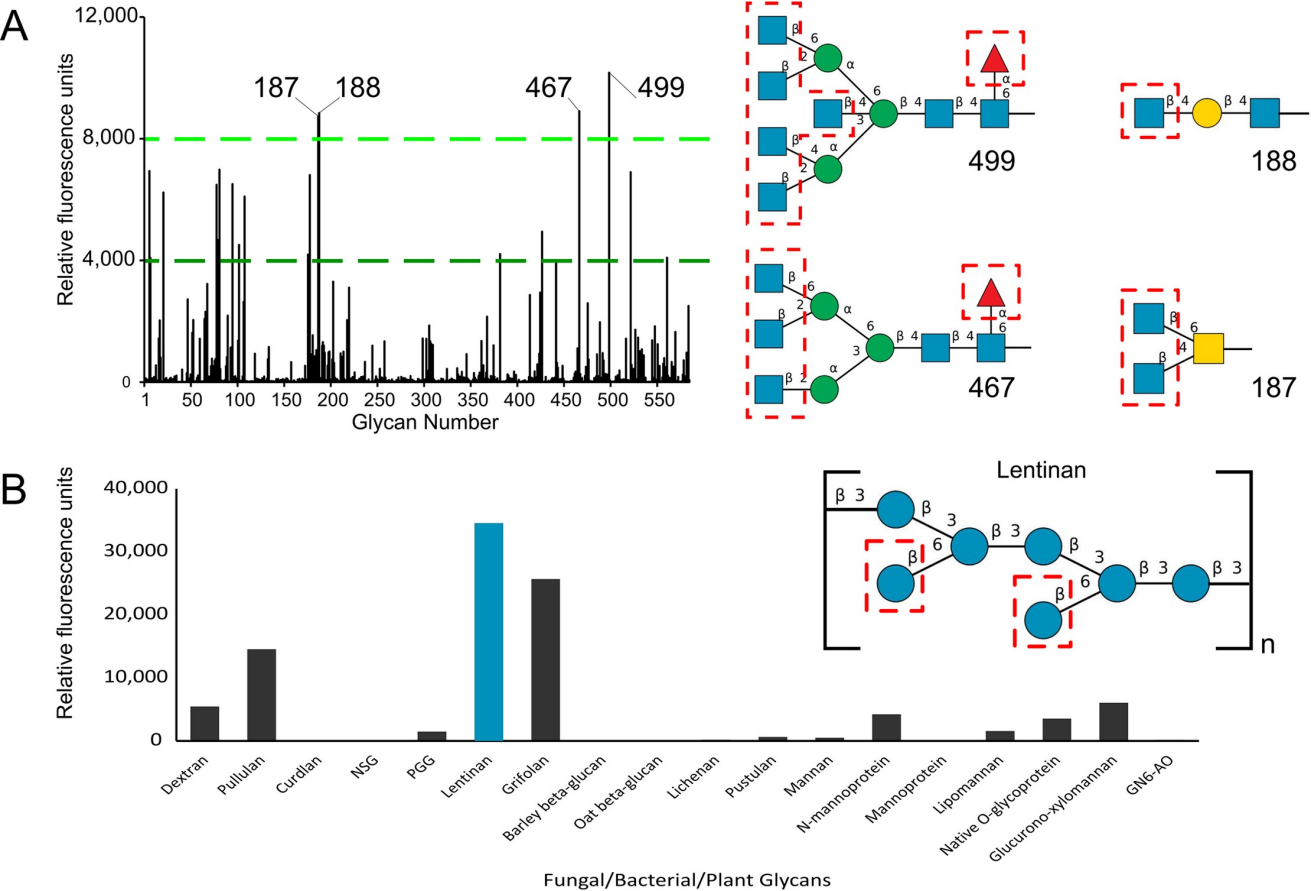
Binding of *Mh*PA14 to Glycan arrays. A) Fluorescent measurements of an array of 585 glycans of varying complexity following incubation with GFP_*Mh*PA14 (200 μg/mL). The fluorescence measured for each glycan is an average of four replicate spots. The four glycan spots that fluoresced above 8000 RFU are labelled, and their structures are presented on the right. Blue squares = N-acetylglucosamine, blue circles = glucose, yellow squares = N-acetylgalactosamine, yellow circles = galactose, green circles = mannose, red triangle = fucose. Terminal sugars proposed to be strong-binders via the competitive assay are outlined in red. B) Fluorescent measurements from a second array, containing eighteen glycans extracted from biological sources following incubation with GFP_*Mh*PA14 (50 ug/mL) and detected through anti-GFP antibody. The fluorescence measured for each glycan is an average of two replicate spots. The highest fluorescing glycan is coloured blue, and its repeating structure is shown on the right using the same colour scheme as in A).

Additionally, the following information is missing from the Acknowledgments: “Finally, we thank Kim Munro from the Protein Function Discovery (PFD) facility at Queen's University for his assistance with CD and ITC.”
