# Supplementary material for: Structure and functional analysis of a bacterial adhesin sugar-binding domain
Source: PLoS One. 2019 Jul 23;14(7):e0220045. doi: 10.1371/journal.pone.0220045 (PMC6650083; doi:10.1371/journal.pone.0220045)
Supplement: S1 Fig — Sequences for the two constructs are shown as combinations of MhPA14 (yellow), GFP (green), and additional vector and linker sequences (grey). (PDF) [file pone.0220045.s001.pdf]

>*Mh*PA14
MASSHHHHHHSSGLVPRGSHMAPDAQADSFGGVAVQGLFGEYYAYAQGSDGGNLSNVAQVKAFIAANEAD
ATFIGRNIDYGSVSGDLGGNGKVQSFLKDDAGSLSTDPENSSDAIVKLTGNLELQAGTYQFRVRADDGYR
IEVNGQTVAEYNGNQGANTRTGSEFTLTGDGPHSVEIVYWDQGGAAQLRIELREQGGAYEIFGSQHASHG
SENP

>GFP_*Mh*PA14
MASSHHHHHHSSGLVPRGSHMVSKGEELFTGVVPILVELDGDVNGHKFSVSGEGEGDATYGKLTLKFICT
TGKLPVPWPTLVTTLTYGVQCFSRYPDHMKQHDFFKSAMPEGYVQERTIFFKDDGNYKTRAEVKFEGDTL
VNRIELKGIDFKEDGNILGHKLEYNYNSHNVYIMADKQKNGIKVNFKIRHNIEDGSVQLADHYQQNTPIG
DGPVLLPDNHYLSTQSALSKDPNEKRDHMVLLEFVTAAGITLGMDELYKGAGHMAPDAQADSFGGVAVQG
LFGEYYAYAQGSDGGNLSNVAQVKAFIAANEADATFIGRNIDYGSVSGDLGGNGKVQSFLKDDAGSLSTD
PENSSDAIVKLTGNLELQAGTYQFRVRADDGYRIEVNGQTVAEYNGNQGANTRTGSEFTLTGDGPHSVEI
VYWDQGGAAQLRIELREQGGAYEIFGSQHASHGSENP
