# Supplementary figures and images for: Structure and functional analysis of a bacterial adhesin sugar-binding domain
Source: PLoS One. 2019 Jul 23;14(7):e0220045. doi: 10.1371/journal.pone.0220045 (PMC6650083; doi:10.1371/journal.pone.0220045)

A

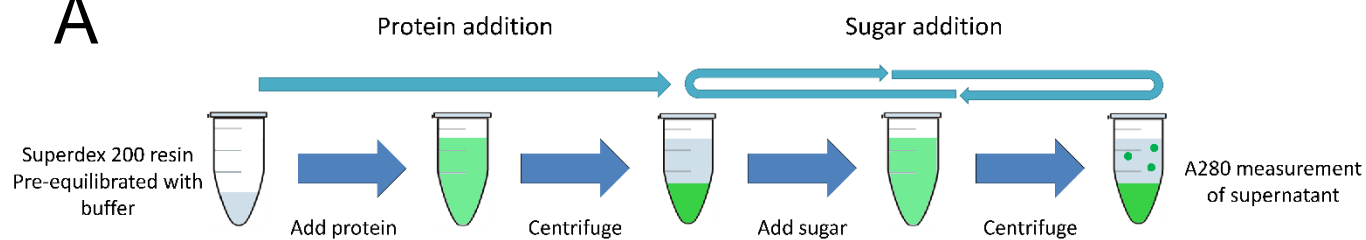

B

## GFP\_*Mh*PA14 Batch Method with S200 Resin

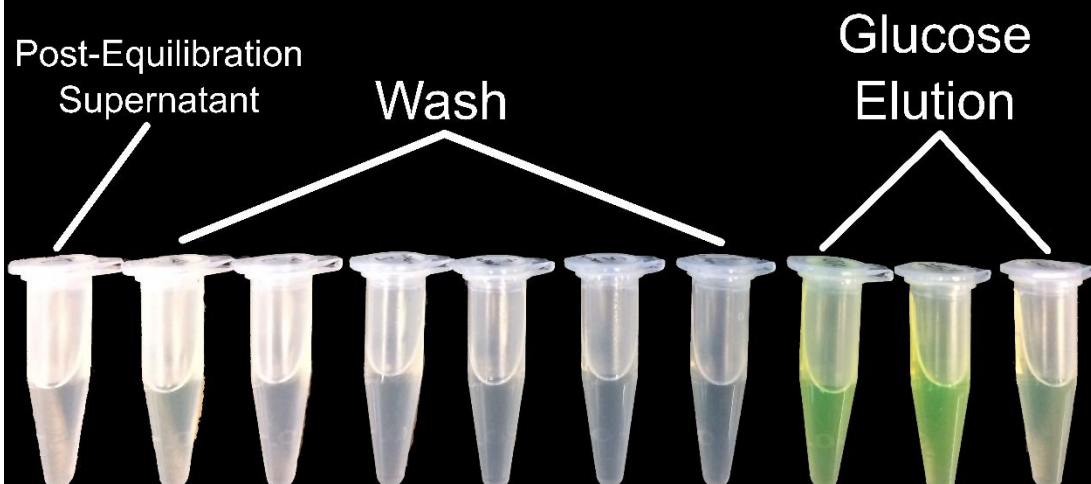

Supplement: S2 Fig — A) Overview of the dextran-affinity assay, showing the process from resin equilibration to repetitive addition of sugars. B) Elution of GFP_MhPA14 from S200 beads in 1.5-mL tubes using glucose. (PDF) [file pone.0220045.s002.pdf]

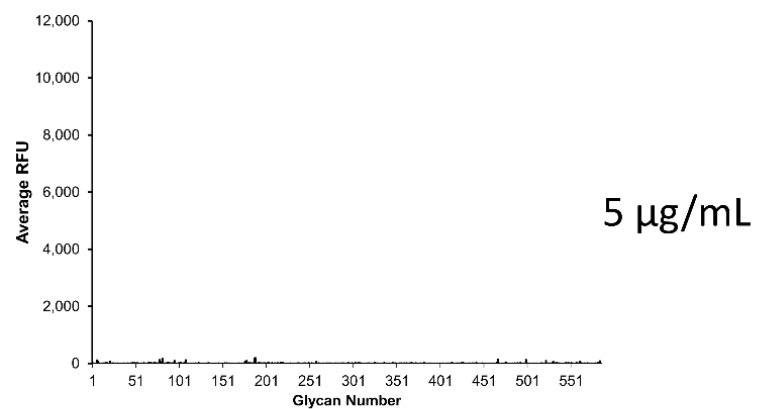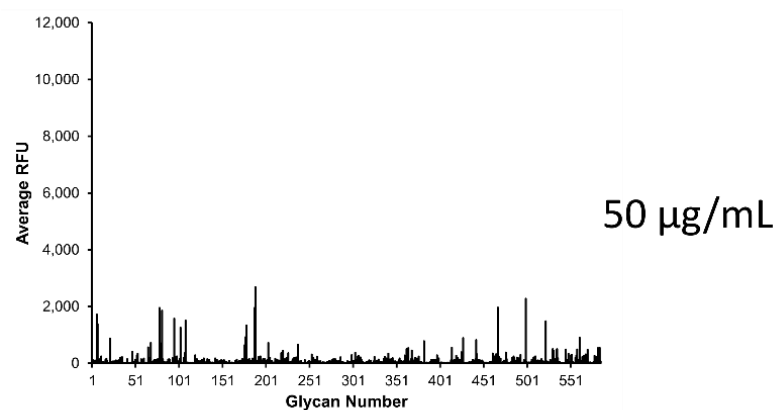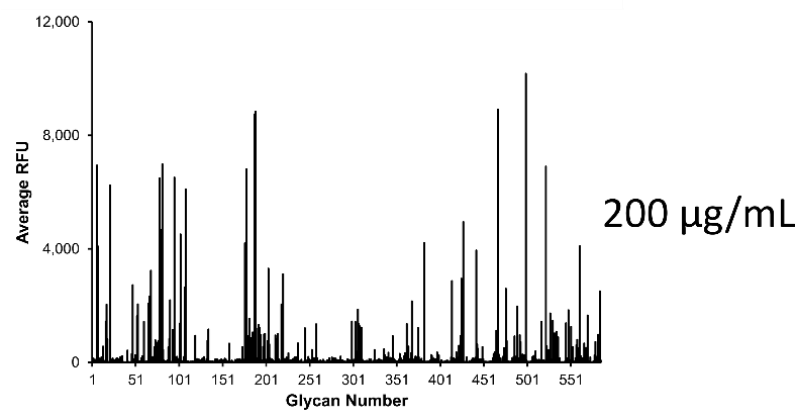

Supplement: S4 Fig — MhPA14 was incubated with the CFG glycan array at three different protein concentrations. The relative fluorescence of each glycan is an average of four replicate spots. (PDF) [file pone.0220045.s004.pdf]
