# Supplementary material for: Structure and functional analysis of a bacterial adhesin sugar-binding domain
Source: PLoS One. 2019 Jul 23;14(7):e0220045. doi: 10.1371/journal.pone.0220045 (PMC6650083; doi:10.1371/journal.pone.0220045)
Supplement: S3 Fig — Glucose moieties are coloured red, galactose moieties are coloured blue, and fucose moieties are colored magenta. Trans equatorial hydroxyls are coloured green. (PDF) [file pone.0220045.s003.pdf]

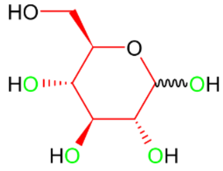

Glucose

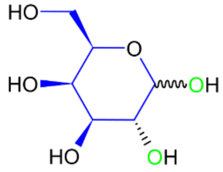

Galactose

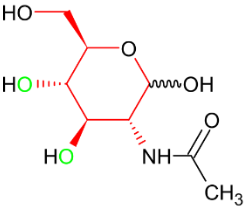

N-acetylglucosamine

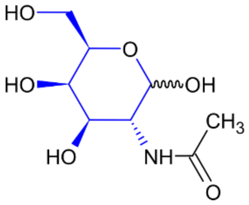

N-acetylgalactosamine

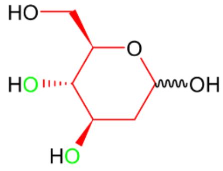

2-Deoxy-D-Glucose

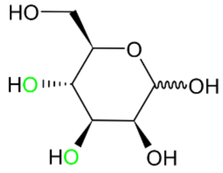

Mannose

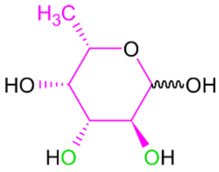

Fucose

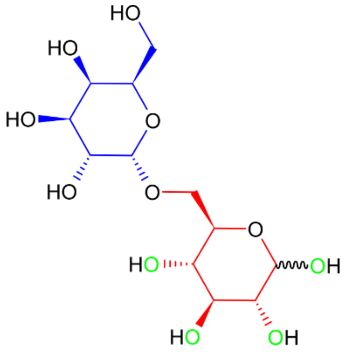

Melibiose

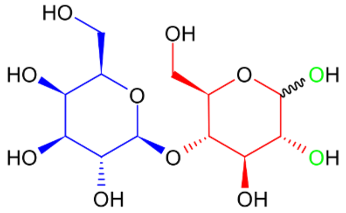

Lactose

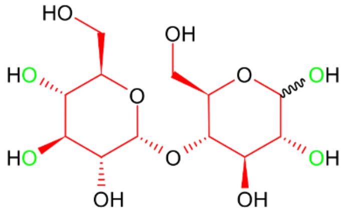

Maltose

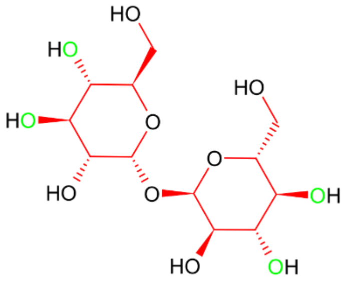

Trehalose

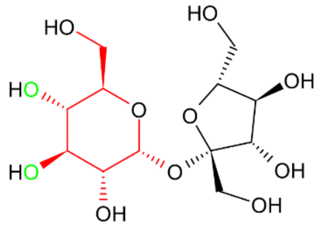

Sucrose
